# Successful *knock-in* of Hypertrophic Cardiomyopathy-mutation R723G into the *MYH7* gene mimics HCM pathology in pigs

**DOI:** 10.1038/s41598-018-22936-z

**Published:** 2018-03-19

**Authors:** J. Montag, B. Petersen, A. K. Flögel, E. Becker, A. Lucas-Hahn, G. J. Cost, C. Mühlfeld, T. Kraft, H. Niemann, B. Brenner

**Affiliations:** 1 Institute for Molecular and Cell Physiology, Hannover Medical School, Carl-Neuberg-Str. 1, 30625 Hannover, Germany; 2 Institute of Farm Animal Genetics, Friedrich-Loeffler-Institut, Hoeltystrasse 10, Mariensee, 31535 Neustadt, Germany; 3 Sangamo Therapeutics, 501 Canal Boulevard, CA 94804 Richmond, USA; 4 Institute of Functional and Applied Anatomy, Hannover Medical School Carl-Neuberg-Str. 1, 30625 Hannover, Germany; 5 REBIRTH, Cluster of Excellence, Hannover Medical School, Hannover, 30625 Germany; 6 Present Address: Casebia Therapeutics, 455 Mission Bay Boulevard South, San Francisco, CA 94158 USA

## Abstract

Familial Hypertrophic Cardiomyopathy (HCM) is the most common inherited cardiac disease. About 30% of the patients are heterozygous for mutations in the *MYH7* gene encoding the ß-myosin heavy chain (MyHC). Hallmarks of HCM are cardiomyocyte disarray and hypertrophy of the left ventricle, the symptoms range from slight arrhythmias to sudden cardiac death or heart failure. To gain insight into the underlying mechanisms of the diseases’ etiology we aimed to generate genome edited pigs with an HCM-mutation. We used TALEN-mediated genome editing and successfully introduced the HCM-point mutation R723G into the *MYH7* gene of porcine fibroblasts and subsequently cloned pigs that were heterozygous for the HCM-mutation R723G. No off-target effects were determined in the R723G-pigs. Surprisingly, the animals died within 24 h *post partem*, probably due to heart failure as indicated by a shift in the a/ß-MyHC ratio in the left ventricle. Most interestingly, the neonatal pigs displayed features of HCM, including mild myocyte disarray, malformed nuclei, and *MYH7*-overexpression. The finding of HCM-specific pathology in neonatal R723G-piglets suggests a very early onset of the disease and highlights the importance of novel large animal models for studying causative mechanisms and long-term progression of human cardiac diseases.

## Introduction

Hypertrophic cardiomyopathy (HCM) is the most common inherited cardiac disease with an incidence of 1:500^1^. Hallmarks of HCM are cardiomyocyte disarray, interstitial fibrosis, and hypertrophy of the left ventricle^2,3^. Disease progression can be variable among affected families and individuals with symptoms ranging from slight arrhythmias to sudden cardiac death or heart failure^4^. Most HCM cases are caused by mutations in genes that encode for sarcomeric proteins^5^. The two most frequently affected genes are the *MYH7* gene that encodes for the ß-myosin heavy chain (ß-MyHC), and the *MYBPC3* gene encoding for cardiac myosin binding protein C^6^. The clinical symptoms become manifest during fetal development for some mutations^7^, while for most patients the HCM phenotype becomes evident during adolescence or adult life^2^. However, the mechanisms that cause the typical HCM pathology are still largely unknown. Direct effects of the mutation on protein function^8^, environmental and genetic factors^9^, and/or disruption of the cardiomyocyte network due to a cell-to-cell expression imbalance of mutant mRNA in individual cardiomyocytes resulting in cell-to-cell functional imbalance^10^ have been proposed to trigger disease progression. In addition, primary effects of mutations in cardiomyocytes of end stage-HCM patients can be altered by secondary adaptations such as changes in phosphorylation of contractile proteins^11,12^.

Cardiac tissue from HCM patients is scarce and tissue samples from children or other family members are not available, thus preventing any longitudinal studies on the time course of the disease. In 1996 the first transgenic mouse model encoding for the HCM mutation R403Q had been reported^13^ and several additional HCM mice have been generated since then. However, these rodent models have a major drawback, especially when it relates to mutations in the ß-MyHC locus. Mice express the fast alpha-myosin in the heart, and orthologous ß-MyHC mutations do not lead to the phenotype typically found in HCM patients^14^. In addition, cardiac electrophysiological properties in mice differ significantly from those in humans. This emphasizes the need for the generation of new large animal models, such as the domestic pig^15,16^. Specifically for cardiovascular diseases, the domestic pig is a useful model because of its great similarities in cardiac anatomy, cardiovascular function, and electrophysiology^17,18^. Moreover, myosin isoform expression in cardiac development is comparable in human and porcine fetuses. The ß-MyHC mRNA and protein are detected in the first part of pregnancy, in the porcine fetus on day 22 post gestation^19^ and week 14 of gestation in humans^20^. In both species, ß-MyHC expression levels increase and α-MyHC expression decreases in the second half of gestation and α-MyHC expression even further decreases after birth^19,20^. In the adult left ventricle, ß-MyHC is the predominant isoform and only low levels of α-MyHC are expressed, 1–10% in humans^21,22^, and 12.5% in pigs^23^. In the failing heart, myosin isoform expression in the left ventricle shifts further towards ß-MyHC, the α-isoform decreases to non-detectable levels^24^.

Recently, designer endonucleases, such as ZFNs, TALEN, CRISPR/Cas9 have emerged as powerful tools for targeted genetic engineering in pigs^17,18,25^. However, the majority of the pigs reported to date have gene *knock-outs*, while genetically modified pigs carrying point mutations and no additional selection marker have only rarely been reported^26^. Here, we report the successful TALEN-guided *knock-in* of the orthologous HCM-mutation R723G in the *MYH7* gene of porcine fetal fibroblasts from German landrace pigs and somatic cell nuclear transfer (SCNT)-based cloning of piglets from these cells. To our knowledge, this is the first report on a selection-free, SCNT-based *knock-in* of a point mutation in pigs.

## Results

### Generation of R723G-genome edited porcine fetal fibroblasts

The point mutation R723G (c.2223C > G, NM_000257) in the ß-MyHC gene *MYH7* causes severe hypertrophic cardiomyopathy in human patients^27–29^. We chose this mutation to generate a large animal model for HCM by introducing the orthologous point mutation (c.2167C > G, NM_213855) in the genome of German Landrace pigs via TALEN based genome editing.

**Figure 1:**
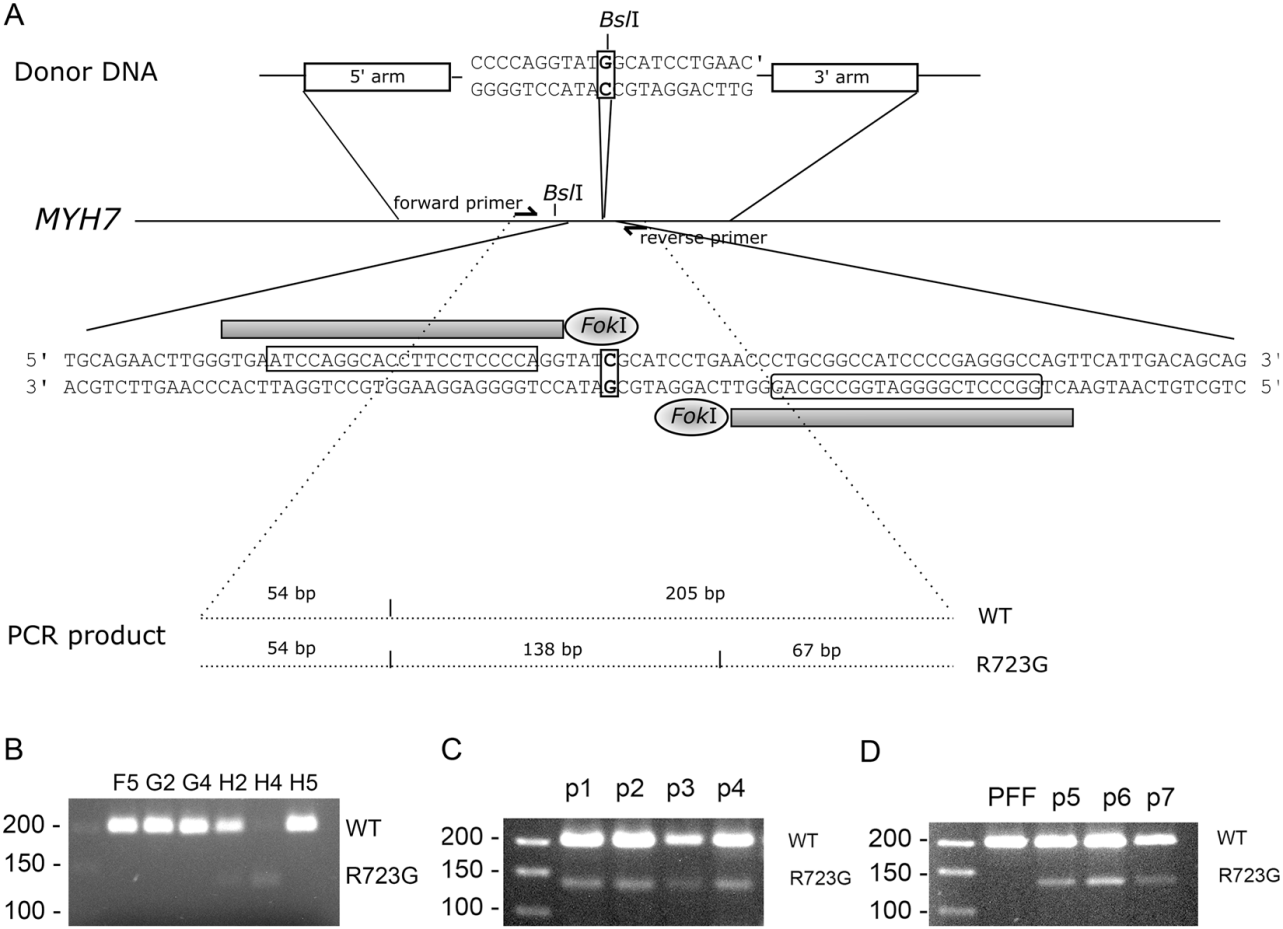
Generation of transgenic fibroblasts and pigs. (**A**) Schematic drawing of the genome editing approach. A donor DNA encoding for the point mutation c.2223 C > G was generated. The point mutation results in an additional *Bsl*I restriction site. A double strand break was introduced in the porcine genomic DNA by TALENs and the point mutation was integrated by homology directed repair. Screening was accomplished by PCR and subsequent *Bsl*I restriction. The wildtype (WT) DNA generates a 205 bp and the genome edited (R723G) DNA a 138 bp fragment. (**B–D**) Agarose gel electrophoresis of *Bsl*I restriction fragments from PCR on DNA from transfected cell cultures (**B**), piglets cloned from cell culture H4 (**C**), and piglets from a subclone of cell culture H4 and the wildtype porcine fetal fibroblasts (PFF) as control (**D**).

The specific TALENs bind 5–26 bp upstream and 3–22 bp downstream of the targeted nucleotide, respectively. The donor DNA was generated for specific introduction of the point mutation. The donor DNA plasmid encodes for homologous arms that range from 500 bp at the 5’ to 250 bp at the 3′ end of the position of the point mutation (Fig. 1A). The 3′-homology arm surpasses a sequence gap in the porcine reference sequence (Sus scrofa 10.2, NC_010449.4, chr.7, 81078606–81078706). This region was determined by Sanger sequencing in German Landrace pigs (Fig. S1).

Porcine fetal fibroblasts were co-transfected with equimolar ratios of vectors encoding for TALENs and donor DNA. Transfected fibroblasts were subcultured and screened for the mutation by PCR amplification of a 259 bp amplicon on the *MYH7* gene covering codon 723. Subsequent mutation-specific *Bsl*I restriction treatment resulted in a 205 bp fragment from the wildtype DNA and a 138 bp fragment from the R723G DNA. Presumably, the cultures contained both, genome edited and wildtype cells and the mutation was integrated into one allele in a certain propotion of the cells. Therefore, we expected wildtype and R723G specific fragments in genome edited cultures. Screening of 31 culture lysates revealed 5 cultures with a restriction fragment of 138 bp (Fig. 1B). Sanger sequencing validated the successful knock-in of the desired point mutation in porcine fibroblasts (Fig. S2).

### Generation of genome edited pigs

Fibroblasts from the subculture with the most prominent R723G per wildtype specific band (H4) were employed in SCNT and reconstructed embryos were transferred to two surrogate sows; one pregnancy was established and delivered four piglets (p1–p4). One animal was stillborn and the three others were weak and did not thrive. *Bsl*I-restriction analysis revealed the mutation-specific 138 bp fragment and the wildtype specific 205 bp fragment in all four animals (Fig. 1C), indicating a successful mono-allelic *knock-in* of the orthologous mutation R723G in the *MYH7* gene in the four piglets.

In a second approach, we produced a clonal porcine fetal fibroblast culture that encodes for the mutation R723G in one allele of the *MYH7* gene. Two pregnancies were terminated at day 27 and six fetuses were harvested for tissue analysis. Fetal fibroblasts were extracted and cultured *in vitro*. *Bsl*I-restriction analysis revealed mutation specific fragments in 2 out of the 6 fetuses. The heterozygous mutation R723G was verified by sequencing (Fig. S3) and fibroblasts from both cultures were subjected to SCNT. Reconstructed embryos were transferred to 16 recipients, of which seven remained pregnant. Altogether 16 piglets were born, eleven were stillborn and five animals were born alive, but did not thrive and were euthanized within 24 h *post partem* for collection of tissue for analysis. In parallel, embryos were cloned from WT-fibroblasts used for TALEN mediated gene editing and three transfers resulted in nine healthy and viable clones, and five stillborn animals (Table 1), indicating that the lack of vitality of R723G piglets was specific for these cells and most likely associated with the HCM-mutation.

**Table 1:** Embryo transfers for cloning of pigs from genome edited fibroblasts and controls.

SCNT donors	No. of recipients (pregnancies)	Number of cloned offspring			
		Total	Stillborn	Postnatal death	Survived
Cell culture H4 (WT and R723G)	2 (1)	4 (R723G)	1	3	0
Cell clone 516/F2B (R723G)	12 (5)	13	8	5	0
Cell clone 516/F3B (R723G)	4 (2)	3	3	0	0
Cell clone 321/F13 (WT)	3 (3)	15	5	0	10

Seven of the R723G-piglets were dissected directly *post mortem*, four from the initial cloning approach (p1–p4, Fig. 1C) and three from the re-cloning (p5-p7, Fig. 1D). Sequence analysis confirmed that all genome edited animals carried the mono-allelic mutation c.2167C > G (Fig. S3).

### Pathological alteration in genome edited piglets

**Figure 2:**
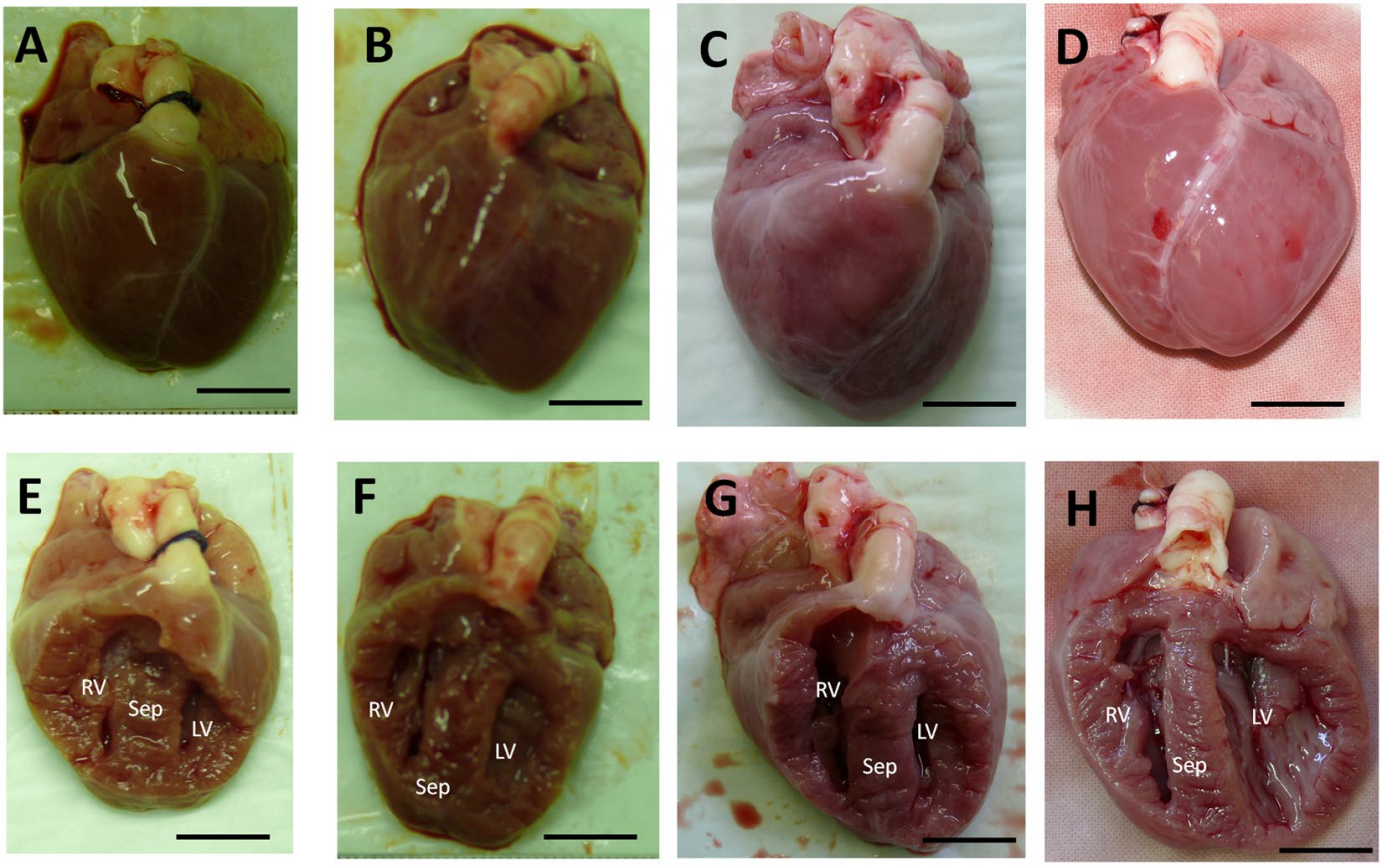
Gross morphology of hearts from genome edited animals. Representative hearts of genome edited (**A–C**) and control (**D**) animals. The animals were male, less than 24 h of age and of comparable birth weight. Coronal sections of the ventricles of genome edited (**E–G**) and control (**H**) animals show that right and left ventricles are comparably sized in each animal as expected for neonates. Scale bar represents 1 cm. RV: right ventricle; LV: left ventricle; Sep: septum.

**Figure 3:**
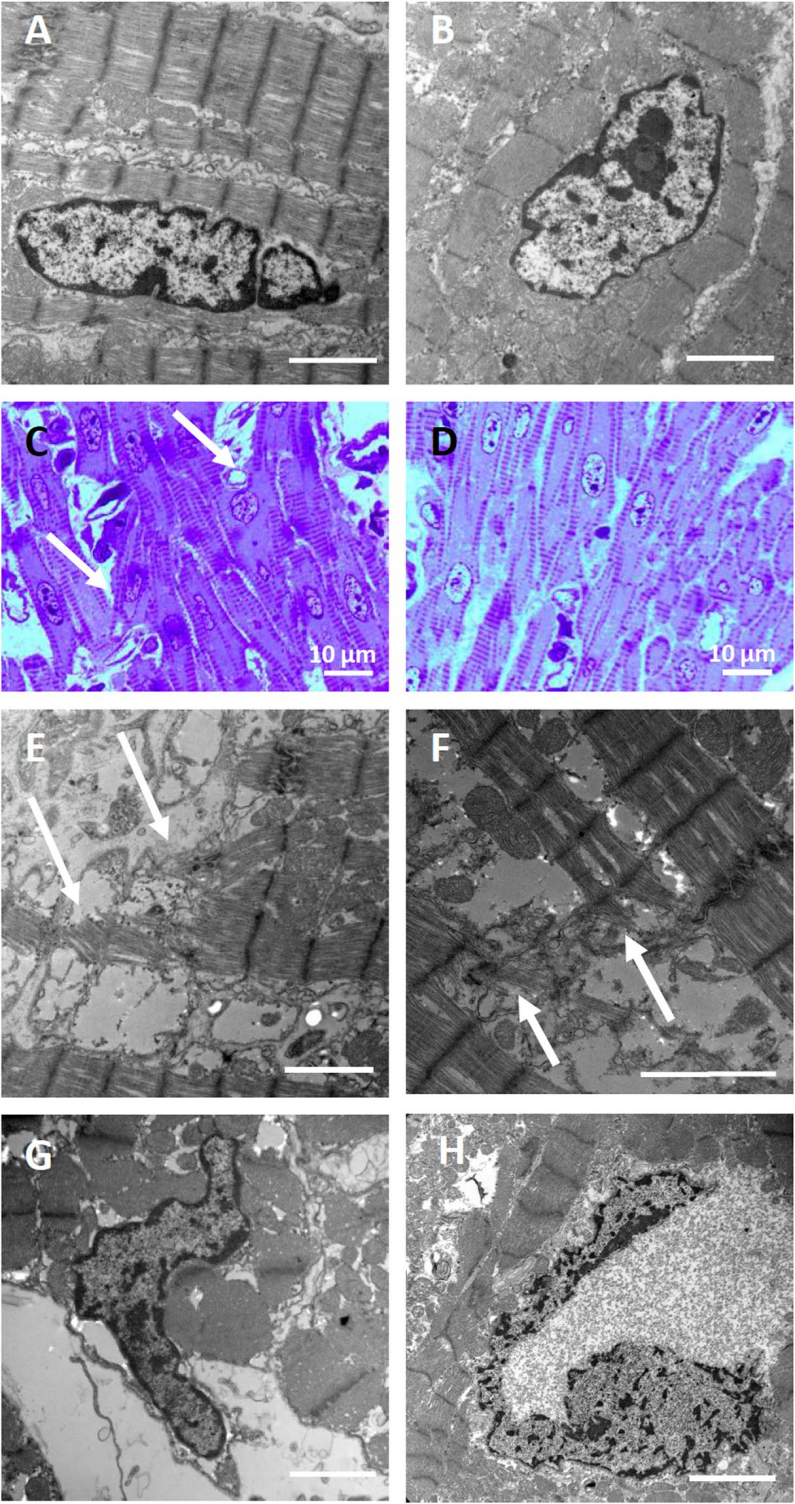
Histopathological analysis of cardiac tissue of the transgenic animals. The myofibrils are mostly regular aligned in the transgenic animals (**A**) and in the wildtype control (**B**). However, signs of myocyte disarray were detected in the transgenic animals (**C**) but not in the controls (**D**) and also mechanically ineffective endings of sarcomeres (**E,F**). Intriguingly, the nuclei of the cardiomyocytes in the transgenic pigs were often malformed (**G**), such nuclei can also be found in human HCM-patients with the same mutation (**H**). The scale bar represents 2 µm unless indicated otherwise.

Analysis of four genome edited animals revealed myositis, caliber variation of muscle fibers and muscle degeneration, cysts in liver and kidney, and abdominal effusions (Table S1). Heart size from genome edited animals (Fig. 2A–C) was comparable to age-and sex-matched controls (Fig. 2D). Also right and left ventricles were equally sized in both, genome edited (Fig. 2E–G) and control (Fig. 2H) animals, as expected for neonates^30^. The hearts showed no obvious hypertrophy (Fig. 2, Tables S1 and S2). Histopathological analysis of cardiac tissue showed mainly well aligned myofibrils (Fig. 3A), similar to the wildtype controls (Fig. 3B). However, a mild form of myocyte disarray – a prominent feature in HCM - was detected in genetically modified animals (Fig. 3C); this was not seen in the controls (Fig. 3D). In addition, mechanically ineffective endings of sarcomeres were detected in R723G hearts (Fig. 3E and F). Nuclei of R723G piglets showed abnormal enlargement (Fig. 3G), similar to malformed nuclei in human HCM-patients with the mutation R723G (Fig. 3H). In addition, the animals also had pathological alterations that are typical for a premortal status such as hyperemia in spleen, lung or kidney. The lung of animals that died within the first 24 h showed edema and ceratin lamella. In animals that died perinatally, the lungs showed dystelectasis, most likely due to incomplete postnatal development. This may also have been the cause of the dilated tubules in kidneys of 3 of the 4 animals, since it had been shown that asphyxia in newborn piglets can lead to tubule dilation^31^. Asphyxia may have occurred in the R723G animals due to insufficient perinatal oxygen delivery. In heart and liver, the animals had vacuoles with glycogen storages that were not found in a 4-day old control. This is most likely due to a rapid decrease in glycogen storages during the first five to six days after birth^32^.

### No off target events were detected in the genome edited animals

To test for potential off-target mutations in genome edited animals, we performed whole genome sequencing. Genomic DNA from a genome edited animal (p7) and from fibroblasts of the same passage that had been used for mutagenesis was sequenced and the sequence reads were compared to the porcine reference sequence (Sus scrofa 10.2) and to known variants (ENSEMBL variant detector). We identified 86 SNPs and 19 indels in the exome of 63 genes of the genome edited animal that were not detected in the fibroblast control (Table S3). Importantly, no variant in coding or non-coding regions of the porcine genome corresponded to the top 100 off-target sites predicted by the DNA-binding assay for the TALEN pair (Table S4).

To test whether the variants were responsible for either the lethality or the other pathological symptoms of the piglets, we performed a database analysis. Each gene was checked for its function using the NCBI gene-database. For non-annotated genes, we used the PANTHER classification system^33^ to assign orthologs, that were subsequently checked in the NCBI gene-database. For two genes, no information was available. Comparison of the function of each gene and potential involvement in disorders revealed no association to either liver, kidney, cardiac or muscular metabolism or disease. A lethal phenotype associated with mutations had been described for the gene SLC35D1. However, no symptom of the associated Schneckenbecken dysplasia was determined in genome edited animals (Table S2), thus we can rule out that this variant caused neonatal death. Notably, 16/63 affected genes encode for olfactory receptors, that are located on different chromosomes and chromosomal loci. This may indicate a conserved region of these receptors that is not detected or falsely annotated in control cells due to a sequencing bias.

### The R723G-allele is expressed at a substantially lower ratio than the WT-allele

To verify expression of the R723G encoding allele, we established a quantitative allele specific mRNA expression assay based on the silent single nucleotide polymorphism (SNP, c.2193C>T). The wildtype sequence was maintained in the C-allele of all genome edited animals (Fig. S4). Total RNA from septum/left ventricular tissue was used in RT-PCR resulting in a 377 bp amplicon. Upon *AvaI* restriction, the C-allele (WT) generated a specific 184 bp fragment, the T-allele (R723G) generated a specific 235 bp fragment and both alleles generated 142 bp fragments (Fig. S4). The *Ava*I treated DNA was separated on an agarose gel. The optical density was determined and normalized to fragment length to correct for the intercalated ethidium bromide. The relative ratios of wildtype and R723G alleles were calculated. The qPCR was established using DNA-standard plasmids in defined ratios^34^ (Fig. S4).

**Figure 4:**
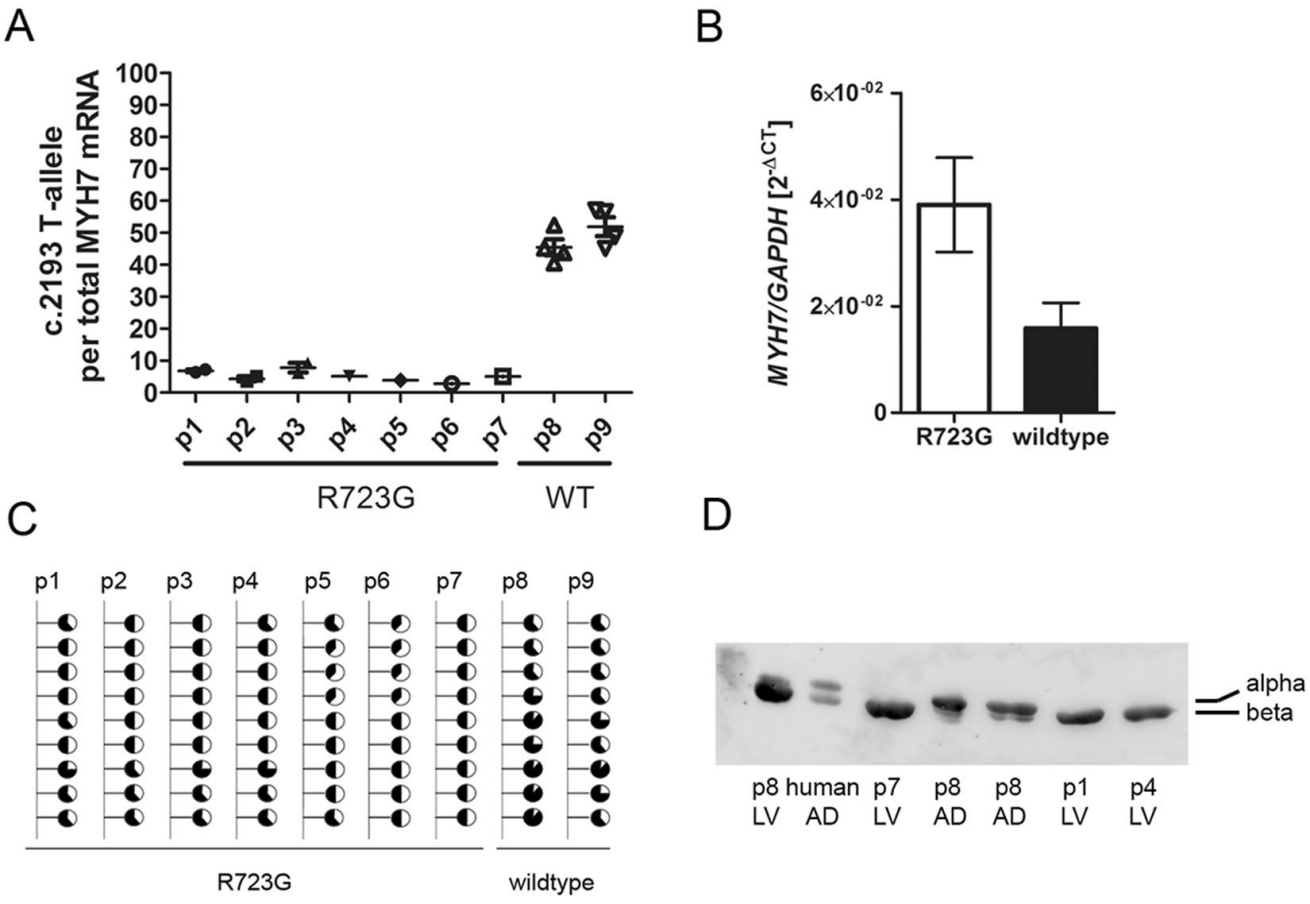
MyHC expression. (**A**) The relative expression of the *MYH7* c.2193T-allele, that harbors the R723G mutation in the genome edited animals (p1–p7) and the wildtype sequence in the wildtype (WT) animals (p8-p9). Quantifications were performed in two independent experiments each in two replicates. (**B**) The relative expression of *MYH7* mRNA per GAPDH was examined by realtime PCR for the genome edited animals and two wildtype controls. Data are presented as the mean and SEM of the 2^−ΔCt^ of the transgenic animals vs. the controls of three independent experiments in duplicates. (**C**) Methylation status of nine CpG sites in the promoter CpG island of the *MYH7* gene in the transgenic animals p1–p7 and two wildtype controls p8-p9. The methylation was assessed by bisulfite specific PCR and analyzed semi-quantitatively from the sequence chromatogram. Filled parts of the circles indicate methylated cytosines and open parts of the circles indicate non-methylated cytosines. (**D**) Western Blot of the α/ß-MyHC isoforms in genome edited piglets (p1, p4, p7) and age- and sex-matched control (p8). Crude protein extracts of porcine left ventricular tissue (LV) were separated by PAGE and transferred to a nitrocellulose membrane. MyHC-isoforms were detected using an α/ß-MyHC-specific antibody. Human and porcine atrium samples (AD) were used as references for α and ß-MyHC isoforms. The α-MyHC shows a higher molecular weight as compared to the ß-MyHC. The full-length blot is presented in Fig. S7.

As expected, we determined fragments specific for both alleles. Unexpectedly, the fraction of the T-allele (R723G) was on average only 5.9% ± 0.4% (mean ± SEM) in the seven genome edited animals. The ratios ranged from 2.3% to 9.8%. We also examined allelic expression of the *MYH7-*mRNA in two age- and sex matched wildtype controls. In these animals both alleles were expressed at comparable ratios; on average we determined 49.2% ± 1.9% for the T-allele (Figs 4A and S4).

To examine whether the low expression of the R723G allele at the mRNA level was also reflected at the protein level, we performed mass spectrometry-based relative quantification of WT and R723G-ß-MyHC protein of four genome edited animals. In the analyzed animals, the R723G-ß-MyHC was below the detection limit of 5% (Fig. S4), suggesting that the fraction of R723G-ß-MyHC is below 5% in the genome edited animals.

### The *MYH7*-gene is overexpressed in genome edited animals

To examine whether the reduced expression of one *MYH7* allele causes reduced total *MYH7* mRNA levels in the cardiac tissue, we quantified *MYH7*-mRNA per *GAPDH*-mRNA in genome edited animals and compared this to two wildtype German landrace pigs. RNA from left ventricular tissue was subjected to real time RT-qPCR. Published primers for the quantification of *GAPDH*^35^ were compared to *MYH7* specific primers to assess efficiency of the amplification process. Both primer pairs had a similar efficacy of amplification (1.00 for *MYH7* and 1.04 for *GAPDH*, Fig. S5). Relative quantification was performed twice in duplicates in two independent replicates and analyzed using the 2^−ΔCT^-method. Intriguingly, the genome edited animals showed an increased relative expression of the *MYH7* gene, on average expression was 2.3 ± 0.2 fold higher when compared to the control animals (Fig. 4B).

### Altered methylation of the *MYH7* promotor in genome edited animals

To test whether the altered expression of the *MYH7* gene was related to changes of the methylation status of the gene, we applied bisulfite sequencing of the *MYH7*-promotor using genomic DNA from genome edited and control animals. Sequence chromatograms were analyzed semi-quantitatively to determine the ratio of cytosins/thymidines at CpG sites as a measure for methylation^36^. In control animals, we determined higher levels of cytosins than thymidines at CpG sites, indicating that CpG sites on both alleles were predominantly methylated rather than non-methylated. In contrast, all seven genome edited animals showed similar levels of methylation at cytosines and thymidines at nearly all analyzed CpG sites (Fig. 4C). This suggests a lower level of total methylation of the *MYH7* promotor in genome edited animals.

### Only ß-MyHC is expressed in the heart of the genome edited animals

In the healthy porcine heart, ß-myosin is the predominant myosin isoform. In addition, 12.5% of alpha myosin are expressed. To determine expression of MyHC-isoforms in cardiac tissue of genome edited animals, we performed SDS-PAGE and western blot analysis using an α/ß-MyHC specific antibody (Fig. 4D). Results were compared to that of age- and sex-matched controls. Samples from atrium of a human HCM patient and a wildtype pig were used as references for α- and ß-MyHC discrimination. Western blotting revealed a single band in the left ventricle (LV) of each genome edited animal (p1, p4, p7 in Fig. 4D) corresponding to the ß-MyHC of the porcine atrium sample (p8, AD). Interestingly, a second, substantially weaker band of slightly higher molecular weight was visible in the left ventricle of the control animal (p8, LV). This weaker band corresponds to the α-MyHC band, as determined in the porcine atrium sample (p8, AD) which was used as internal standard for the MyHC isoforms.

## Discussion

HCM is the most common inherited cardiac disease. Even though numerous mutations in different sarcomeric proteins, e.g. the ß-MyHC, have been identified that are causative for HCM, the underlying pathomechanisms remain elusive^9^. Specifically, onset and long term progression of HCM cannot be studied in human patients^16^. Rodent models do not fully display the human HCM phenotype, mainly due to substantially different physiology. In addition, rodents express a different cardiac myosin isoform. ß-MyHC mutations in the cardiac alpha-MyHC, which constitutes the major myosin isoform in the mouse, do not reflect the phenotype of the human mutation^14^. Domestic pigs are widely used animal models for human cardiac research because they have a similar cardiac anatomy and have many cardiovascular and electrophysiological similarities with humans^17,18^. In the last years, designer endonucleases such as zinc finger nucleases, TALENs and the CRISPR/Cas9 system have emerged as new tools in genome editing in many biological systems, thus facilitating efficient generation of large animal models carrying mutations causative for human diseases. However, the majority of genome edited pigs reported to date are knock-out animals, only few pigs with a functional knock-in have been reported^18^.

To generate a suitable large animal model for HCM, we introduced the orthologous HCM-mutation R723G into the *MYH7* gene of the porcine genome. The challenge was to achieve single base substitution without introducing additional selection markers into the *MYH7* gene. To date two different approaches have been successfully used to generate genome edited pigs. Nucleases and donor DNA are either injected into the cytoplasm of zygotes or transfected into fibroblasts which are subsequently used in somatic cell nuclear transfer (SCNT). The first method yields higher efficacy for homology directed repair (HDR), and has been used for a knock-in of a point mutation without a selection marker in pigs; but it turned out that most animals had chimeric genotypes^26^. In contrast, HDR-driven genetic modification in fibroblasts provides lower efficacy, but screening for modified cells is significantly facilitated. The majority of the reports on point mutations has also introduced selection markers into the genome to facilitate screening^18^; fibroblasts with point mutations have not yet been used for SCNT-based cloning^37^.

Here we report the first successful SCNT-based generation of selection marker-free genome edited pigs carrying a point mutation. Using a TALEN-guided approach we have successfully generated genetically modified fibroblasts that encode for the point mutation c.2167C>G (R723G) in the porcine *MYH7-*gene. SCNT-based cloning of these cells resulted in genome edited piglets that were heterozygous for the HCM-point mutation. No alterations in the presumptive top 100 off-target sites of the TALEN pair were detected, suggesting that no off-target events had occurred during genome editing.

All animals died perinatally or within 24 h *post partem*. Even though the mutation R723G causes a severe form of HCM with early disease onset and high incidence of heart failure and sudden cardiac death in humans^27–29^, perinatal death was not reported. We have no evidence that genome editing or the cloning protocol itself may have caused the observed mortality. Wildtype controls cloned from the identical cell line used for genome editing, showed 67% viable and 90% healthy clones, which is in the normal range for SCNT-cloned animals^38^. Thus, neither the cloning process, nor the genetic background of the fibroblasts affected the viability of cloned animals. However, the wildtype cells were cultivated separately from their genome edited counterparts which had been passaged after transfection with TALENs. This may have contributed to exonic alterations that were found in 63 genes in the genome edited animal. However, literature and data base research on the affected genes revealed no association to lethality or to piglets’ pathophysiology. Comparable ratios of exon alterations have been reported for double-strand break induced mutagenesis in pluripotent stem cells without effects on the basic characteristics of the cells^39^. Nevertheless, we cannot completely rule out that one of the determined variants may have indirectly affected viability of the piglets.

We propose that the early death of the piglets may be associated with perturbed expression of the *MYH7*-gene and of MyHC-isoforms in the R723G-piglets as compared to age- and sex-matched wildtype controls. We identified a marked allelic imbalance of the *MYH7-*alleles, the R723G-allele was expressed at very low levels. Importantly, the low expression of the R723G allele in the piglets did not lead to haploinsufficiency, as reported for *MYBPC3* mutations of human HCM patients^40^. In contrast, total *MYH7* mRNA was 2.3 fold over-expressed in R723G piglets. In addition, we found that the *MYH7* promoter was hypomethylated in genome edited pigs. Recent studies have shown that reduced methylation of the *MYH7*-promoter is directly associated with an increased *MYH7*-expression^41,42^. Since the mutated allele was expressed at very low ratios in genome edited animals, we hypothesize that the wildtype allele is hypomethylated to compensate for the low level of the mutant allele and by overcompensation finally causing *MYH7*-overexpression. It should be noted, that in mouse models of cardiac hypertrophy, a 2-fold overexpression of the *MYH7*-gene is considered to be an early marker of hypertrophic development^43^. Upregulated *MYH7* has also been determined in human patients^44,45^.

Apart from the *MYH7*-overexpression, we found that the α-MyHC isoform was not expressed in the left ventricle of R723G-piglets. In contrast, α-MyHC was detected at low levels in age- and sex matched control animals, as it is characteristic for healthy human and porcine left ventricular tissue^22,23^. Interestingly, a decrease in α-MyHC has been reported in the left ventricle of failing hearts in different cardiac diseases and is assumed to be associated with the transition from hypertrophy to heart failure^24^. The reduction of α-MyHC isoform strongly supports our notion of heart failure in the neonatal animals with mutation R723G. This conclusion is further supported by edema in the lungs of animals that died postnatally^46^. Similarly human patients with the mutation R723G show a high incidence of heart failure at the end stage of the disease^27–29^ which may reflect a transition from hypertrophy to heart failure in the course of disease development^47^.

In heart failure diagnosis, liver cysts and ascites have been determined as non-cardiac findings^46^. The abdominal effusions and the cysts in liver and kidney observed in the R723G-piglets may thus be secondary effects of the cardiac dysfunction. However, we cannot completely rule out that the cyst-formation may also be associated with another - yet undetermined - disease. However, the major genes involved in polycystic kidney and liver diseases such as *PKD1, KD2, SEC. 63, PRKCSH*, and *PKHD1* did not show any sequence variants in the R723G-piglets. Interestingly, in human neonates with fetal HCM, abdominal effusions^7^ and liver- and kidney-disorders^48^ have been observed.

The animals showed also mild pathological features that are found more severely in HCM-patients. We discovered signs of myocyte disarray and mechanically ineffective sarcomere endings in cardiac tissue, which may be indicative for the onset of myofibrillar loss, a key feature of HCM^3^. In severely affected human R723G patients, extreme myocyte disorganization is thought to be responsible for reduced force output of cardiomyocytes^11^. In the R723G piglets, calcium sensitivity, maximum active force, as well as passive force and cross-bridge cycling kinetics as indicated by k_tr_ [rate constant for force redevelopment after a quick slackening and re-stretch of the cardiomyocytes^11^] were unchanged compared to control animals (Fig. S6). This most likely is due to the low expression level of the mutated myosin in the myocardium of the piglets. In human patients the mutant allele makes up ~70% of the total *MYH7* mRNA and protein^10,34,49^, and is associated with severe alterations in force generation and myocyte disarray^11^. In the R723G piglets, heart weight and septum thickness were not increased and myofibrils were mainly regularly aligned, consistent with mild HCM-pathology. Besides the observed myocyte disarray, the cardiomyocytes of the R723-piglets showed enlarged and malformed nuclei, which are also found in human HCM patients^50^ and that we have also determined in human R723G patients. In addition, caliber variations of skeletal muscle fibers and muscle degeneration in the R723G-piglets are comparable to the variable fiber size variations and necrotic fibers that have been reported in HCM patients with mutations in the *MYH7*-gene^51^.

Our combined findings indicate that our genome edited R723G piglets show indications of an HCM phenotype, even though these signs are not very prominent. The early death of the piglets suggests a severe form of prenatal development of HCM due to mutation R723G in the pig. Such an early disease development warrants further investigation with this pig model. Interestingly, in a mouse model with inducible mutant α-MyHC it was found that expression of the mutant myosin in early postnatal periods strongly triggers HCM development^52^. Yet, to overcome the limitations of the early death, further genome edited pigs with other mutations in *MYH7* need to be generated. Particularly, porcine models with inducible mutant ß-MyHC may provide increased viability and enable longitudinal studies of HCM pathology. In mouse models expressing mutation R403Q in the endogenous α-MyHC it was shown that the HCM phenotype is strongly age-dependent^53^.

Most mouse models express HCM-related human ß-MyHC mutations either in the α-MyHC^13,54–56^ or in transgenic α-MyHC from rat^57^ or mouse^58^. These animals show HCM pathology such as left ventricular hypertrophy, reduced cardiac function and cardiomyocyte disarray. Yet, due to the fast α-MyHC isoform in the murine heart which in humans is only found at very low levels in the ventricles, direct translation of the effects in mouse heart to human heart pathology is difficult^9^. In addition, cardiac muscle strips and mutated α-MyHC isolated from such mouse models have been used to examine functional effects of myosin mutations like alterations of calcium-sensitivity of isometric force and cross-bridge kinetics^55,59^. Since it was shown that the α- vs. ß-MyHC isoform strongly influences the effects of specific mutations on myosin function^14^, mutation effects in murine α-MyHC cannot directly be related to functional alterations in human ß-MyHC. Therefore, the generation of genome edited pigs with mutations in the ventricular slow ß-MyHC is of great interest, since this will allow to study ß-MyHC-mutation effects in animals with cardiovascular physiology similar to humans.

A surprising finding in our pig model was the low expression of mutant mRNA and protein in the hearts of the genome edited piglets. However, in transgenic mice expressing rat α-MyHC with the R403Q mutation, despite a very low expression of mutant myosin of only 1–12% of total heart myosin, HCM histopathology similar to human hearts was detected^57^. This corroborates our finding that low levels of mutant MyHC-mRNA and protein in the pigs can induce features of HCM pathology and heart failure, pointing to a dominant negative effect of the mutant protein.

In summary, we report here the successful knock-in of the HCM-associated point mutation R723G in the porcine *MYH7*-gene and the generation of genetically modified HCM-piglets. This is the first genome edited pig model for human cardiovascular disease generated by a selection-free SCNT-based introduction of a point mutation. The young age of the animals, and pathological features that are indicative for HCM suggest that the RG723G piglets displayed an early stage of HCM. In humans with the R723G-mutation, clinical symptoms become manifest at an age of >12 years^27–29^, although HCM related pathology must have started much earlier. Our findings suggest for the piglets a fetal onset of the R723G-associated HCM-pathology, leading to early heart failure and perinatal death.

## Material and Methods

### Generation of TALENs and Donor DNA

The TALEN pair SBS 101820/SBS 101821 was generated according to the method of Miller *et al*.^60^. SBS 101820 binds to the DNA sequence 5′-CCAGGCACCTTCCTCCC-3′; SBS 101821 binds to the DNA sequence 5′-GGCCGCAGGGTTCAGG-3′. Off-target cleavage sites were predicted using a SELEX-weighted search of the *Sus scrofa* genome as described^61^. Genomic DNA of a German Landrace pig was extracted from whole blood using the DNA Mini Blood Kit (Qiagen, Germany) according to the suppliers′ instructions. A part of the *MYH7* gene was amplified using the donor DNA primer set and cloned into the pGEM-T vector (Promega, Germany). The R723G encoding base pair exchange was introduced via site-directed mutagenesis according to published protocols^62^. Homologous arms according to 501 bp upstream and 252 bp downstream of the point mutation were subcloned into the pGEM3-Zf(+) vector.

### Porcine fetal fibroblasts and somatic cloning

Primary porcine fetal fibroblast cultures were established from a male fetus at day 25 post conception and maintained for six passages as described previously^63^. For genome editing, 1.3 × 10^6^ cells were resuspended in 800 µl of electroporation buffer (Bio-Rad, Germany), supplemented with 7.5 µg each, TALEN plasmids and donor DNA plasmid. Fibroblasts were transfected using the Gene Pulser XcelTM (Bio-Rad, Germany) at 250 V/400 µF for 30 ms. A total of 10.000 cells each were cultured for 24 h at 37 °C, followed by incubation at 30 °C for five days, and four passages at 37 °C. An aliquot of the cells was subjected to mutation screening. SCNT was performed with cells from mutation positive cultures as described previously^63^. Two pregnancies were terminated at day 25, and fetal fibroblasts were generated from genome edited fetuses and used in SCNT. The pregnancies were allowed to go to term. Genome edited piglets and two male, 1 day old wildtype animals were euthanized for tissue collection. The animals were dissected immediately *post mortem*, and hearts were perfused using Custodiol (Dr. Franz Köhler Chemie GmbH, Germany). Cardiac tissue was flash-frozen for subsequent RNA, protein and force-pCa measurements or immersed in 1.5% GA and 1.5% PFA in 0.15 M HEPES buffer or 4% PFA for histopathological analysis.

### PCR

The PCR reaction mix consisted of 1 × reaction buffer S, 12.5 mM MgCl_2_, 5 pmol forward primer, 5 pmol reverse primer, 5 nmol dNTPs each and 1 u Taq (PeqLab, Germany) in a final volume of 25 µl. The solution was amplified by denaturation at 95 °C for 30 sec, annealing according to the respective annealing temperatures given in (Table S5) for 30 sec and elongation at 72 °C for 30 sec for 45 cycles. Subsequently, the target DNA was elongated at 72 °C for 2 min. PCR-amplicons for RNA-quantification spanned 3 exons to discriminate between mRNA and potential gDNA-contamination.

### Screening for R723G-genome edited DNA

A total of 1 × 10^5^ porcine fetal fibroblasts collected at passage 4 post-transfection were washed with PBS, resuspended in 20 µl cell lysis buffer (Ambion, USA) and lysed at 75 °C for 10 min. One µl of the lysate or 10 ng gDNA were subjected to PCR. A total of 9 µl of the PCR product was incubated with 1 µl *Bsl*I for 2 h at 37 °C. *Bsl*I restriction resulted in a 205 bp and a 56 bp fragment for the wildtype and a 138 bp, a 67 bp and a 56 bp fragment for the R723G DNA. Restriction fragments were analyzed by agarose electrophoresis.

### Sequence analysis

PCR products were purified using the NucleoSpin Gel and PCR clean up kit (Macherey Nagel, Germany) according to the suppliers’ instructions. Thirty ng/100 bp were subjected to Sanger sequencing. Sanger sequencing was performed by SeqLab (Göttingen, Germany). Whole Genome Sequencing from genomic DNA of the genome edited animal p7 and the wildtype porcine fetal fibroblast cell line was performed by GATC (Konstanz, Germany) using the Illumina platform. The sequences were aligned to the *Sus scrofa* 10.2 assembly and to the ESEMBL variant detector.

### Histopathological analysis of genome edited piglets

Four genome edited piglets were subjected to pathological analysis at the Institute of Pathology of the University of Veterinary Medicine Hannover (Hannover, Germany). For electron microscopy, samples were immersed in a mixture of 1.5% glutaraldehyde/1.5% paraformaldehyde in 0.15 M HEPES buffer and were then incubated with 1% osmium tetroxide, half-saturated uranyl acetate and dehydrated in an ascending acetone series. After embedding in epoxy resins, the samples were subjected to ultramicrotomy and semi- and ultrathin sections were generated and either stained with toluidine blue (semithin sections for light microscopy) or post-stained with uranyl acetate and lead citrate (ultrathin sections for electron microscopy). Electron microscopy was performed using a Morgagni transmission electron microscope (FEI, Eindhoven, Netherlands).

### RNA extraction and cDNA-synthesis

Shock frozen septum/left ventricular tissue of the genome edited piglets and of age- and sex matched controls were ground under liquid nitrogen. RNA was extracted using the PeqGold total RNA kit according to the suppliers’ instructions and subsequently reverse transcribed in cDNA reaction mix (1x RT-buffer, 10 pmol RT-primer or oligo dT primer (Table S1), respectively, 2.5 nmol dNTPs each, 0.5 µl RiboSafe, and 50 u Tetro RT (Bioline)) in a final volume of 20 µl for 1 h at 42 °C.

### Allele specific myosin expression analysis

For quantification of allele specific expression, cDNA was subjected to PCR amplification followed by reconditioning PCR^34,64^. A total of 9 µl of reconditioned PCR product and 1 µl *Sty*I-HF were incubated for 1 h at 37 °C, resulting in a 142 bp fragment from both alleles, a 184 bp fragment from the wildtype and a 235 bp fragment from the R723G allele. Restriction fragments were separated on 3% sieving agarose (Biozym) gels. The integrated optical densities (IOD) of the respective bands were analyzed using the software Origin^TM^ (Microcal^TM^ Software) by curve fitting of the intensity profiles generated by the Total Lab X1 software. The IODs were normalized to the length of the fragment. The relative allelic mRNA expression was calculated as the ratio of wildtype or the R723G-fragment per common fragment, respectively.

Protein isoform quantification was performed by mass spectrometry as described previously for the human R723G mutation^49^.

### Relative quantification of total *MYH7-*mRNA

One µl of oligo dT-cDNA was subjected to real time-PCR reaction mix (1X SYBR Green PCR Master Mix, 2 pmol each of forward primer and reverse primer for *MYH7* or GAPDH, respectively (Table S1)) in a final volume of 20 µl in duplicates. After an initial denaturation, amplification was performed at 95 °C for 10 min in 40 successive cycles of 15 sec at 95 °C and 45 sec at 60 °C in a 7500 Real Time PCR System (Applied Biosystems, USA). Data were analyzed according to the 2^−ΔCT^-method^65^.

### Methylation analysis of the *MYH7*-gene

CpG-island detection in the *MYH7* promotor and Bisulfite specific primer design was accomplished using the software Methyl Primer express. Genomic DNA was subjected to bisulfite conversion using the EpiMark Bisulfite Conversion Kit (NEB, USA) according to the suppliers’ instructions and subsequently the *MYH7-*promotor was PCR amplified. PCR-products were subjected to Sanger sequencing and methylation status of each CpG site was assessed semi-quantitatively from the sequence chromatograms^36^.

### Western Blot analysis of porcine α and ß-MyHC

Left ventricular tissue from the genome edited animals and age- and sex-matched controls were ground under liquid nitrogen and then diluted in RotiLoad1 buffer (Roth, Germany) at a final concentration of 40 µg/µl of total protein. Tissue was lysed by incubation for 1 h at room temperature and 4 min at 80 °C. Proteins were separated by PAGE (collection gel: 4% acrylamide/bisacrylamide, 0.4 M EDTA, 0.4% SDS and 70 mM Tris, separating gel: 8% acrylamide/bisacrylamide, 30% glycerol, 0.4% SDS, 0.1 M glycin and 0.2 M Tris-base) at 230 V, 20 mM and 20 W for 48 h at 4 °C). The myosin was subsequently blotted to a 0.22 µm nitrocellulose membrane (GE, USA) for 1,5 h at 30 V in Tris/Glycin-transfer buffer with 10% methanol. For α- and ß-MyHC detection, the membrane was washed with TBS, blocked over night with 3% milk powder non- fat (Santa Cruz, USA) in TBS, incubated with the primary antibody ab50967 (abcam, USA) for 2 h at room temperature, washed in TBS, incubated with the secondary antibody anti-mouse-HRP (BioRad, Germany) for 1 h at room temperature and washed again with TBS. Antibody staining was detected using SuperSignal West Dura (Thermo Fisher Scientific, Germany) in the LAS-system (GE, USA). To discriminate between the α- and ß-MyHC, atrium samples from porcine and human tissue that express both isoforms were used.

### Ethics statement

All experiments were performed according to the German law for animal welfare and approved by an external ethics committee (Nds. Landesamt für Verbraucherschutz und Lebensmittelsicherheit, LAVES AZ 33.14–42502–04–12/0789). Analysis of human samples was approved by the Ethics Committee of Hannover Medical School (No. 2276–2014). Informed consent was obtained according to approved Ethics Committee protocols of the institutions involved. The investigations conformed to the principles of the Declaration of Helsinki^66^.

### Data availability

The authors declare that the data supporting the findings of this study are available within the article and its Supplementary Information files.

## Electronic supplementary material

Supplemental material
