# Supplementary material for: Successful knock-in of Hypertrophic Cardiomyopathy-mutation R723G into the MYH7 gene mimics HCM pathology in pigs
Source: Sci Rep. 2018 Mar 19;8:4786. doi: 10.1038/s41598-018-22936-z (PMC5859159; doi:10.1038/s41598-018-22936-z)
Supplement: Supplementary file 1 — Supplemental material [file 41598_2018_22936_MOESM1_ESM.pdf]

Successful *knock-in* of Hypertrophic Cardiomyopathy-mutation R723G into the *MYH7* gene mimics HCM pathology in pigs

Montag J, Petersen B, Flögel AK, Becker E, Lucas-Hahn A, Cost GJ, Mühlfeld C, Kraft T, Niemann H, Brenner B

SUPPLEMENTARY MATERIAL

Supplementary Tables

Table S1: Pathological phenotype of genome edited piglets

	p10	p11	p12	p13	Potential cause
**death**	perinatal	postnatal	perinatal	postnatal	
**Body weight**	1.18 kg	1.12 kg	0.93 kg	0.66 kg	
**Limbs**	myositis**, muscle degeneration, muscle fiber caliber variation	caliber variation	No data	No data	*MYH7* mutation [1]
**tongue**	inflammation, central muscle degeneration	No data	edema, caliber variation	edema	*MYH7* mutation [1]
**Lung**	hyperemia dyselectase	partial dyselectase, alveolar edema	atelectase	dyselectase, intraalveolar meconium and ceratine lamella	edema: heart failure [2]
**Liver**	vacuolization (lipid storage)	vacuolization (lipid storage), cysts	cysts	vacuolization (glycogen storage)	neonatal glycogen and lipid storage [3] Cysts: fetal HCM [4]
**Kidney**	cysts	cysts	cysts	No data	heart failure [2], fetal HCM [4]
**lymph nodes**	lymphadenitis	lymphadenitis	No data	ulcerous inflammation	*
**Bone marrow**	no abnormal diagnosis	red	red	red	*
**Heart**	No abnormal diagnosis				
**Additional findings**	Abdominal effusion				heart failure [2], fetal HCM [5]

* Reaction to undefined immunologic stimulation, most probably not related to animals' death

** It should be noted that nothing indicates to a toxic insult that may otherwise have caused the myositis in the neonatal animals.

Table S2: Cardiac size* of genome edited animals [mm]

animal	Body weight	Heart height	Heart width	Septum thickness
**p5 (R723G)**	No data	40	31	8
**p6 (R723G)**	No data	41	31	8
**p7 (R723G)**	No data	39	33	8
**p8 (WT)**	1.5 kg	50	44	7
**p9 (WT)**	0.5 kg	34	27	7

*Heart height was measured from the cardiac apex to the aortic root. Heart width and septum thickness refer to the widest site for each heart.

Table S3: Genes with variants in the genome edited animals

	SYMBOL/ID	Gene	Modification	Function
1	SETSIP	ENSSSCG00000005656	base substitution	nucleosome assembly
2	F1RWS0	ENSSSCG00000005796	base substitution	olfactory receptor activity
3	I3LFV8	ENSSSCG00000024409	base substitution	olfactory receptor activity
4	F1RWQ3	ENSSSCG00000005817	base substitution	olfactory receptor activity
5	I3LBT9	ENSSSCG00000028938	base substitution	olfactory receptor activity
6	F1RV62	ENSSSCG00000005872	base substitution	olfactory receptor activity
7	PPAG3	ENSSSCG00000013096	base substitution	pregnancy-associated glycoprotein 3, aspartic proteinase expressed in trophectoderm
8	I3LJT8	ENSSSCG00000028404	base substitution	olfactory receptor activity
9	F1RH28	ENSSSCG00000014530	base substitution	olfactory receptor activity
10	F1S6T5	ENSSSCG00000013412	base substitution	transcription co-repressor activity
11	F1S6J0	ENSSSCG00000023319	base substitution	olfactory receptor activity
12	I3LKX0	ENSSSCG00000029505	base substitution	olfactory receptor activity
13	F1S9Z9	ENSSSCG00000013842	base substitution	heme binding
14	I3L8I3	ENSSSCG00000029101	base substitution	olfactory receptor activity
15	F1SCB1	ENSSSCG00000013809	base substitution	olfactory receptor activity
16	I3LF13	ENSSSCG00000024741	base substitution	no pathyway information available, no ortholog assigned
17	SEMA6A	ENSSSCG00000014224	base substitution	required for proper development of the thalamocortical projection
18	PCDHA13	ENSSSCG00000028030	base substitution	establishment and function of specific cell-cell connections in the brain
19	OR5D18	ENSSSCG00000014458	base substitution	olfactory receptor family
20	LOC100526160	ENSSSCG00000024147	base substitution	olfactory receptor 4C46-like
21	LOC100524490	ENSSSCG00000023055	base substitution	olfactory receptor 2AE1-like
22	MOSPD3	ENSSSCG00000023255	base substitution	motile sperm domain containing 3
23	HN1L	ENSSSCG00000008019	base substitution	hematological and neurological expressed 1-like
24	TCF7L1	ENSSSCG00000008241	base substitution	transcription factor 7 like 1
25	-	ENSSSCG00000008671	base substitution	The human ortholog family proteasome subunit beta type-5 cleaves peptides in an ATP/ubiquitin-dependent

				process in a non-lysosomal pathway
26	RCSD1	ENSSSCG00000006307	base substitution	may regulate the ability of F-actin-capping protein to remodel actin filament assembly (skeletal muscle contraction)
27	OTUD7B	ENSSSCG00000006663	base substitution	deubiquitinase, dysfunction associated to cancer
28	NACA	ENSSSCG00000020830	base substitution	Prevents inappropriate targeting of non-secretory polypeptides to the endoplasmic reticulum
29	HSBP1	ENSSSCG00000000460	base substitution	heat shock factor binding protein 1 interacts with the active trimeric state of HSF1 to negatively regulate HSF1 DNA-binding activity
30	STK38L (NDR2)	ENSSSCG00000022288	base substitution	serine/threonine kinase 38 like, cell cycle progression
31	PABPC4	ENSSSCG00000003660	base substitution	poly(A) binding protein cytoplasmic 4, might be necessary for regulation of stability of labile mRNA species in activated T cells
32	TMP-CH242-74M17.2	ENSSSCG00000001229	base substitution	The human ortholog family proteasome subunit beta type-5 cleaves peptides in an ATP/ubiquitin-dependent process in a non-lysosomal pathway
33	LOC100153899	ENSSSCG00000030371	base substitution	serpin A3-8, involved in hemostasis
34	-	ENSSSCG00000030059	base substitution	olfactory receptor
35	LOC106504562	ENSSSCG00000002624	base substitution	ortholog to Glutathione S-transferase A3 is involved in cellular defense against toxic, carcinogenic, and pharmacologically active electrophilic compounds
36	-	ENSSSCG00000014894	base substitution	human ortholog (Teneurin 4) is involved in neural development
37	USP2	ENSSSCG00000015120	base substitution	deubiquitinates polyubiquitinated target

				proteins such as MDM2, MDM4 and CCND1, influence on circadian rhythm
38	RNF2	ENSSSCG00000015568	base substitution	suppress the activity of, transcription factor CP2 (TFCP2/CP2). Involved in in cell proliferation in early development.
39	SOX9	ENSSSCG00000017251	base substitution	SRY (sex determining region Y)-box 9
40	VPS26A	ENSSSCG00000010250	base substitution	retromer complex component A
41	C10orf76	ENSSSCG00000010571	base substitution	interacts with SEPT3 and 9, unknown function, also for human orthologue
42	CCNYL1	ENSSSCG00000024696	base substitution	spermatogenesis and sperm motility
43	IKZF2	ENSSSCG00000016164	base substitution	negative regulation of transcription from RNA polymerase II promoter
44	RICTOR	ENSSSCG00000016853	base substitution	Subunit of mTORC2, which regulates cell growth and survival in response to hormonal signals. Plays an essential role in embryonic growth and development.
45	F1SBM9	ENSSSCG00000007038	base substitution	uncharacterized protein, orthologous to LEM-domain family (LEM-domain proteins are involved in diverse cellular processes including replication and cell cycle control, chromatin organization and nuclear assembly, the regulation of gene expression and signaling pathways, as well as retroviral infection)
46	I3LD58	ENSSSCG00000024507	base substitution	uncharacterized protein, orthologous to LEM-domain family (LEM-domain proteins are involved in diverse cellular processes including replication and cell cycle control, chromatin organization and nuclear assembly, the

				regulation of gene expression and signaling pathways, as well as retroviral infection)
47	I3L5Z7	ENSSSCG00000022771	base substitution	human orthologue GPR107 is involved in Golgi-to-ER retrograde transport
48	PNKD	ENSSSCG00000029329	base substitution	Mutations in this gene have been associated with the movement disorder paroxysmal non-kinesigenic dyskinesia
49	DGKQ	ENSSSCG00000027286	base substitution	mediates the regeneration of phosphatidylinositol (PI) from diacylglycerol in the PI-cycle during cell signal transduction
50	CYP19A1	ENSSSCG00000030168	indel	cytochrome P450 19A1, involved in sperm fertility regulation
51	-	ENSSSCG00000026788	indel	olfactory receptor
52	FZD7	ENSSSCG00000016111	indel	frizzled class receptro 7, receptors for Wnt signaling proteins
53	CD44	ENSSSCG00000013297	indel	involved in oozyte maturation
54	PTPRS	ENSSSCG00000028612	indel	Receptor-type tyrosine-protein phosphatase S, human and mouse orthologs suggested to be involved in cell-cell interaction, primary axonogenesis, and axon guidance during embryogenesis and implicated in the molecular control of adult nerve repair.
55	-	ENSSSCG00000014380	indel	human ortholog PCDHB15 is involved in ectoderm development
56	LOC100519082	ENSSSCG00000014565	indel	interferon-induced transmembrane protein 1, involved in B cell receptor signalling pathway

57	LOC100627004	ENSSSCG00000028736	indel	interferon-induced transmembrane protein 1-like
58	LY6H	ENSSSCG00000006961	indel	lymphocyte antigen 6 family member H, posttranscriptional modification if GPI-anchored proteins
59	CA8	ENSSSCG00000006233	indel	carbonic anhydrase 8, nitrogen metabolism
60	SYMPK	ENSSSCG00000003093	indel	symplekin functions in the regulation of polyadenylation and promotes gene expression
61	SLC35D1	ENSSSCG00000003802	indel	solute carrier family 35 member D1, UDP-N-acetylglucosamine/UDP-glucose/GDP-mannose transporter may participate in glucuronidation and/or chondroitin sulfate biosynthesis. Mutations in this gene are associated with Schneckenbecken dysplasia* (symptoms:
62	-	ENSSSCG00000029865	indel	assigned to familiy of metabotropic glutamate receptor 5 that may be involved in the regulation of neural network activity and synaptic plasticity
63	OAF	ENSSSCG00000026647	indel	out at first homolog, no function assigned, Diseases associated with OAF include Chronic Maxillary Sinusitis

* Schneckenbecken dysplasia is a neonatally lethal chondroplasia. Patients show “short, broad long-bones with dumbbell-like appearance, flat and hypoplastic vertebral bodies, short and wide fibula, and precocious ossification of the tarsus.” (Borochowitz et al., 1986). Note that the genome edited animals show no signs of dysplasia or any malformation of the bones. Heterozygous knock out mice show no phenotype, whereas homozygous knock-outs die neonatally (Hiraoka et al., 2007).

Table S4: Predicted off-target sites of the TALEN pair used for genome editing

	Chromosome	Start	End	Mismatch
**Target 1***	chr7	81078817	81078866	2
**Target 2**	chr5	72122334	72122395	5
**Target 3**	chr10	25916449	25916509	7
**Target 4**	chr9	1,26E+08	1,26E+08	7
**Target 5**	chr15	1,27E+08	1,27E+08	8
**Target 6**	chr3	19333272	19333325	8
**Target 7**	chr13	58049063	58049116	8
**Target 8**	chr13	57902270	57902323	8
**Target 9**	chr11	80975743	80975802	7
**Target 10**	chr14	1,41E+08	1,41E+08	8
**Target 11**	chr3	1,36E+08	1,36E+08	8
**Target 12**	chr1	2,49E+08	2,49E+08	8
**Target 13**	chr1	2,49E+08	2,49E+08	8
**Target 14**	chr5	1,1E+08	1,1E+08	8
**Target 15**	chr5	56089986	56090046	8
**Target 16**	chr14	1,32E+08	1,32E+08	8
**Target 17**	chr15	3226481	3226539	7
**Target 18**	chr12	21923763	21923824	8
**Target 19**	chr7	62624663	62624712	6
**Target 20**	chr1	1,57E+08	1,57E+08	8
**Target 21**	chr5	66377844	66377899	8
**Target 22**	chr8	1,42E+08	1,42E+08	8
**Target 23**	chr15	65341152	65341206	9
**Target 24**	chr15	65251904	65251958	9
**Target 25**	chrX	92541175	92541232	8
**Target 26**	chr9	1,14E+08	1,14E+08	9
**Target 27**	chr3	37191323	37191383	8
**Target 28**	chr15	16971212	16971272	8
**Target 29**	chr1	1,08E+08	1,08E+08	7
**Target 30**	chr3	1E+08	1E+08	9
**Target 31**	chr15	1,35E+08	1,35E+08	6
**Target 32**	chr6	71093917	71093973	7
**Target 33**	chr10	49559347	49559407	9
**Target 34**	chr10	34092117	34092170	9
**Target 35**	chr16	58052767	58052818	9
**Target 36**	chr3	1,09E+08	1,09E+08	8
**Target 37**	chr7	15706117	15706167	8
**Target 38**	chr3	1,29E+08	1,29E+08	8
**Target 39**	chr8	73656431	73656483	9
**Target 40**	chr10	12555269	12555317	8
**Target 41**	chr9	76471254	76471304	9
**Target 42**	chr13	1,22E+08	1,22E+08	8
**Target 43**	chr1	87380802	87380854	7
**Target 44**	chr2	87468643	87468693	8
**Target 45**	chr13	1,41E+08	1,41E+08	8

**Target 46**	chr10	27801214	27801264	8
**Target 47**	chr1	71888738	71888788	8
**Target 48**	chr13	2,18E+08	2,18E+08	9
**Target 49**	chr8	81264267	81264326	9
**Target 50**	chr15	11393419	11393478	8
**Target 51**	chr6	13053122	13053175	9
**Target 52**	chr17	16191859	16191910	9
**Target 53**	chr17	14405632	14405683	9
**Target 54**	chr7	1,31E+08	1,31E+08	8
**Target 55**	chr13	57588549	57588601	8
**Target 56**	chr3	16315905	16315965	6
**Target 57**	chr3	18579492	18579545	7
**Target 58**	chr3	27152758	27152815	8
**Target 59**	chrX	1,25E+08	1,25E+08	10
**Target 60**	chr14	61902657	61902713	8
**Target 61**	chr8	4835019	4835068	9
**Target 62**	chr1	50320654	50320706	7
**Target 63**	chr8	1,05E+08	1,05E+08	9
**Target 64**	chr2	46988756	46988805	10
**Target 65**	chr14	98213389	98213447	9
**Target 66**	chr15	1,5E+08	1,5E+08	9
**Target 67**	chr15	1,48E+08	1,48E+08	9
**Target 68**	chr2	1,61E+08	1,61E+08	9
**Target 69**	chrX	88606848	88606907	9
**Target 70**	chr2	1,49E+08	1,49E+08	7
**Target 71**	chr9	55092038	55092089	8
**Target 72**	chr2	1,42E+08	1,42E+08	8
**Target 73**	chr7	1,34E+08	1,34E+08	9
**Target 74**	chr3	1,15E+08	1,15E+08	7
**Target 75**	chr11	72797123	72797183	9
**Target 76**	chr3	37357920	37357971	10
**Target 77**	chr10	47204006	47204059	9
**Target 78**	chr14	1,01E+08	1,01E+08	9
**Target 79**	chr3	90916440	90916499	9
**Target 80**	chr4	7098167	7098228	9
**Target 81**	chr5	48586015	48586074	9
**Target 82**	chr7	1,07E+08	1,07E+08	9
**Target 83**	chr8	88109952	88110011	9
**Target 84**	chr9	2962426	2962486	9
**Target 85**	chr17	19192208	19192268	9
**Target 86**	chr7	1,03E+08	1,03E+08	7
**Target 87**	chr8	31705484	31705544	9
**Target 88**	chr9	1,05E+08	1,05E+08	9
**Target 89**	chr1	1,04E+08	1,04E+08	10
**Target 90**	chr11	32668464	32668518	9
**Target 91**	chr1	1,12E+08	1,12E+08	8
**Target 92**	chr6	1,52E+08	1,52E+08	9

**Target 93**	chr4	84313677	84313729	9
**Target 94**	chr14	40790693	40790744	7
**Target 95**	chr5	20884737	20884797	8
**Target 96**	chr18	18786847	18786901	7
**Target 97**	chr13	49530564	49530623	8
**Target 98**	chr17	45670405	45670454	10
**Target 99**	chr16	15047956	15048016	8
**Target 100**	chr15	6665713	6665771	9

***** Target 1 is the *MYH7* gene, the two mismatches represent a CA-insertion, that is not included in the reference sequence NC_010449 but was sequence verified in the German Landrace pigs (see also Fig. S1)

Table S5: PCR- and sequencing primers

PCR	Forward primer	Reverse primer	Amplicon length [bp]	Annealing temperature [°C]
Donor DNA	Intron 18 for<br>AGGTCACCTCCTGATCTCC	Intron 20 rev<br>TCCCCAGTAAGGGCTAGCA	1008	62°C
mutagenesis	723 mut sense<br>TTCCTCCCCAGGTATGGCA	723 mut as<br>AGGGTTCAGGATGCCATAC		60°C
Mutation screening	Ssc719for<br>GTGCTGGAGGGCATCCGC	Ssc719rev<br>CTTCTCTGCTCCTTTCCTG	259	62°C
**Quantification of *MYH7* allele expression**				
cDNA		ATGCGGGTGATGATGCGG		
qPCR	ssc723mRNA-for_StyI<br>TCTATGGGGACTTCCGGCA	ssc719Quant_rev<br>CAGGGGTTTGATCTTGAAG	377	68°C
**Relative quantification of *MYH7* per GAPDH mRNA expression**				
MYH7	Ssc453for<br>TGGCCAAGGCCGTGTATGA	Ssc453rev<br>GAAGATCTCAAAGCCAGCC	125	60°C
GAPDH[6]	ACACTCACTCTTCTACCTTT	CAAATTCATTGTCGTACCA		60°C
**Bisulfite specific PCR and Sequencing**				
	TAGGTTTATTTGGTGGGAG	AACTCCAAAACCAATTCTC	302	56°C

Supplementary figures:

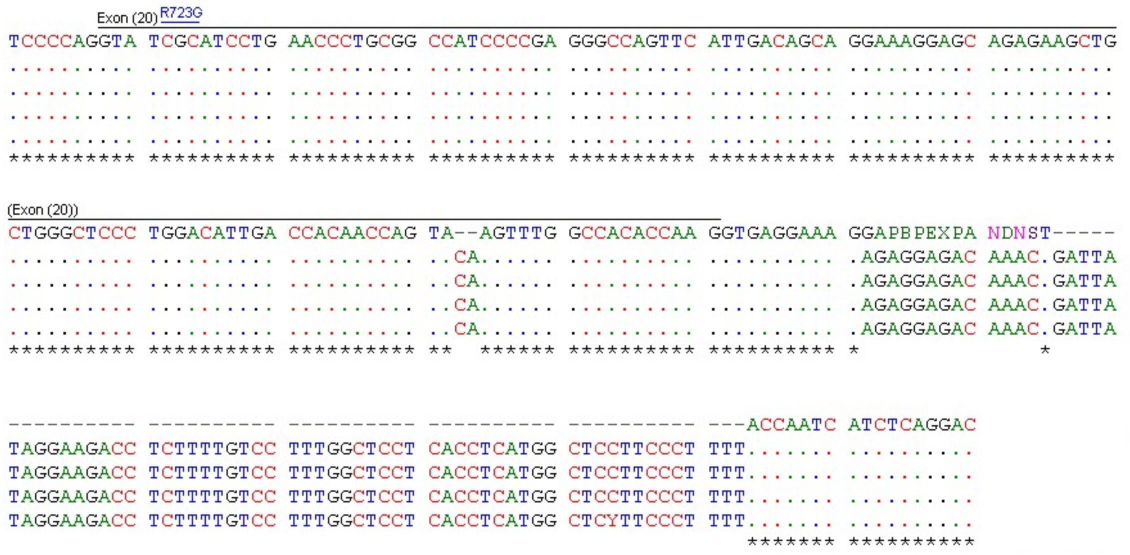

Fig. S1: Sequencing of gap in porcine *MYH7* reference sequence NC_010449. The reference sequence of the *MYH7* gene (NC_010449 alignment sus scrofa 10.2) contains a gap at position 81078606 of estimated 100 bp (row 1). We have sequenced this region in four German Landrace pigs (rows 2-5) by Sanger sequencing of PCR products generated by primers flanking the region. We determined a 72 bp sequence. In addition, we found a CA-insertion 26 bp downstream of the gap. However, these nucleotides are present in the cDNA NM_000257.2, therefore we assume this is a common sequence of the *sus scrofa* species.

A

Culture H4 (BslI positive)

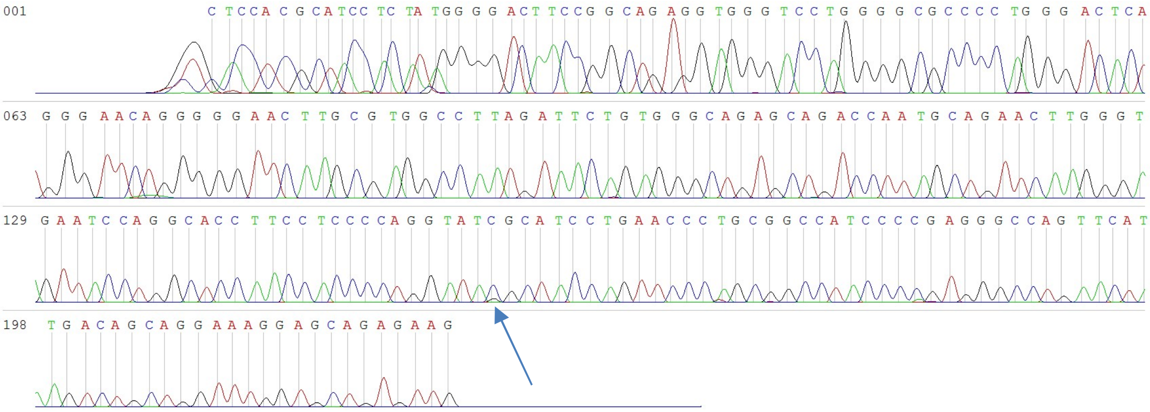

Culture F4 (*BslI* negative)

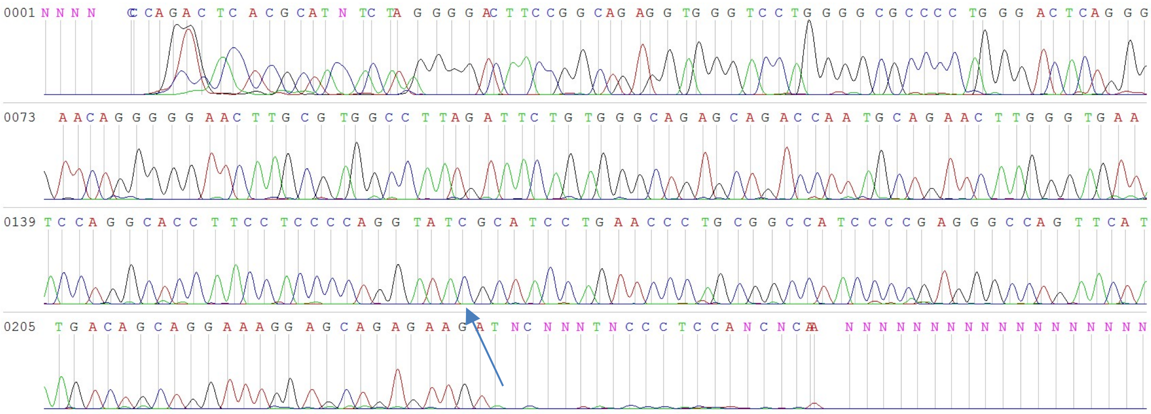

Figure S2: Sanger Sequencing of mutation locus in genome edited fibroblast cultures Porcine fetal fibroblasts were transfected with the TALEN vectors and the DonorDNA encoding vector, separated in subcultures at 10,000 cells per well and cultivated for 4 passages. An aliquot of the cells was lysed, the mutation locus in the *MYH7* gene was PCR amplified and subjected to mutation specific restriction analysis. The PCR product from the restriction analysis-positive culture H4 was analyzed by Sanger sequencing using the PCR-forward primer. Successful genome editing was indicated by the base substitution C>G indicated by the arrow. Two additional polymorphisms can be determined downstream of the mutation locus, however, these are also present in the wildtype fibroblasts (F4).

Clone 516/F2

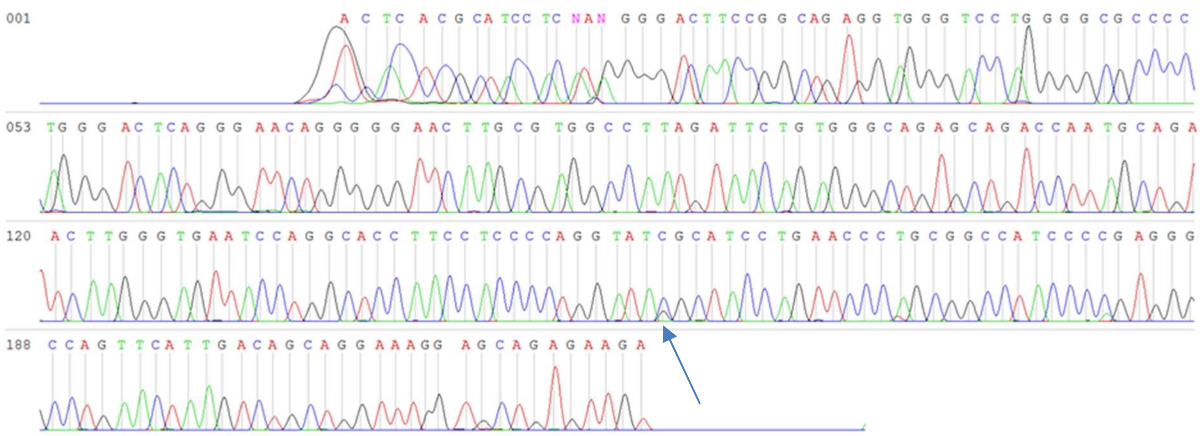

Clone 516/F3

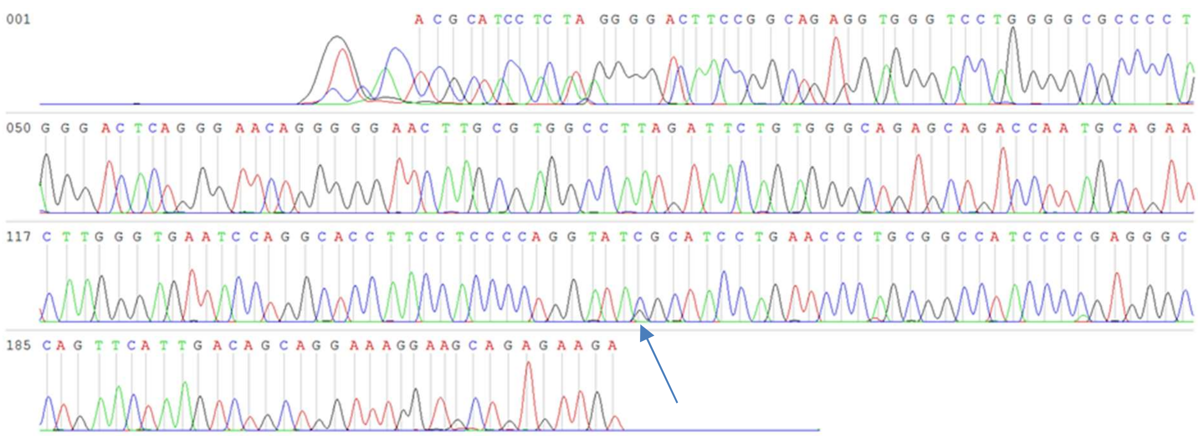

Genome edited pigs:

p1 **p2** **p3** **p4** **p5** **p6** **p7**

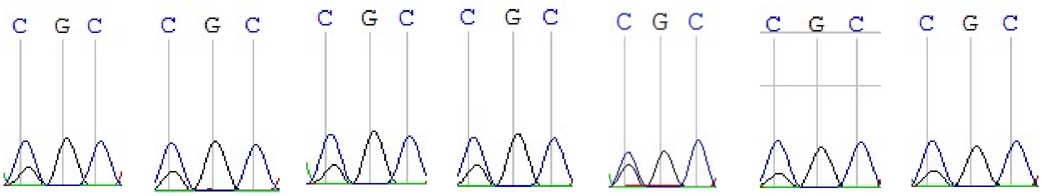

Figure S3: Sanger Sequencing of mutation locus in genome edited clones Culture H4 was used for SCNT, oocytes were transferred to two sows, fibroblasts were isolated from six fetuses and analyzed for the mutation. The two restriction analysis-positive clones 516/F2 and 516/F3 were validated by Sanger sequencing. The position of base substitution is indicated by an arrow. In addition, genomic DNA from cardiac tissue of seven genome edited piglets (p1-p7) was subjected to *MYH7*-specific PCR and codon 723 was analyzed by Sanger sequencing to determine the mono-allelic C>G base substitution.

A

p1

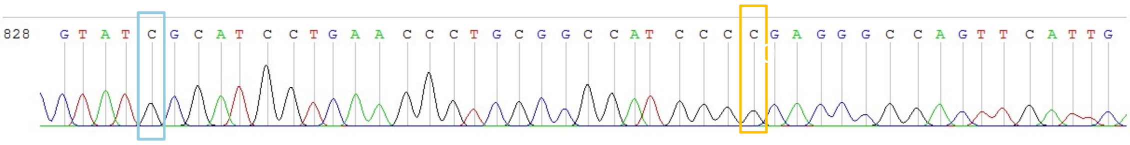

p2

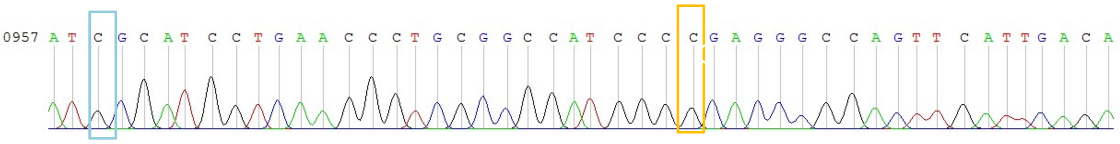

p3

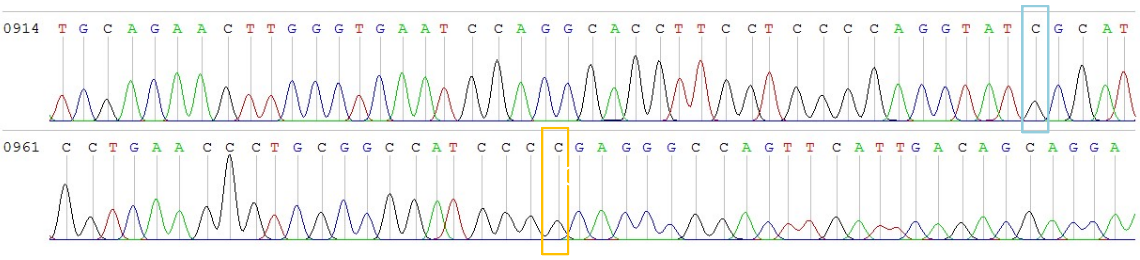

p4

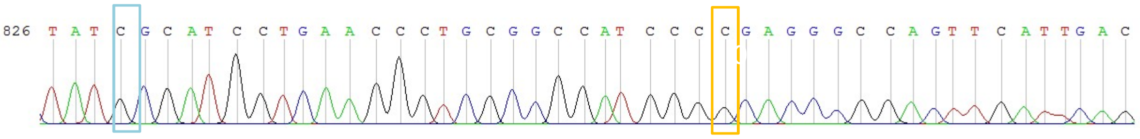

p5

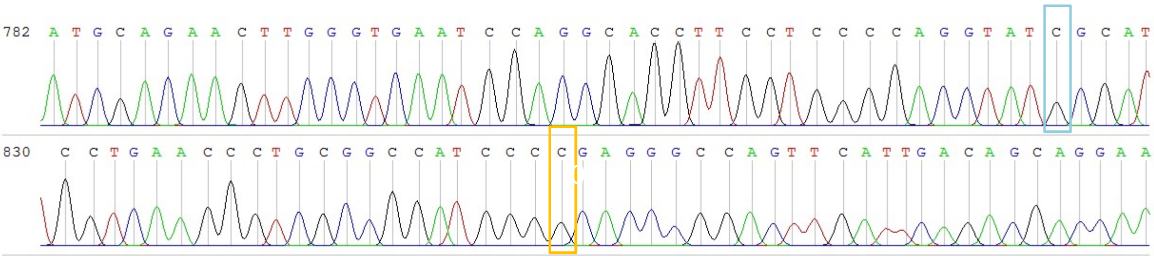

p6

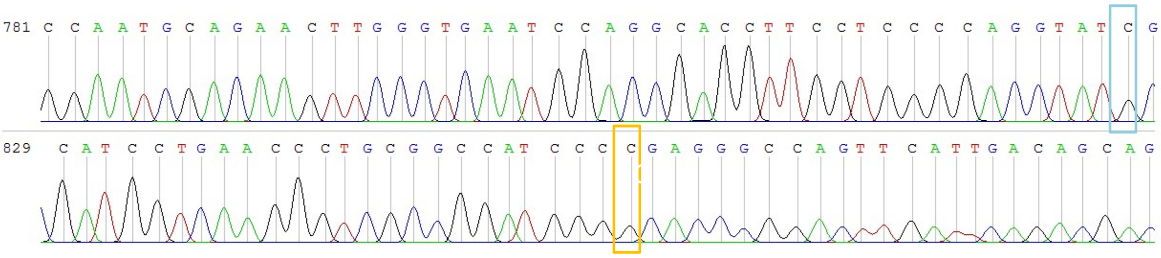

p7

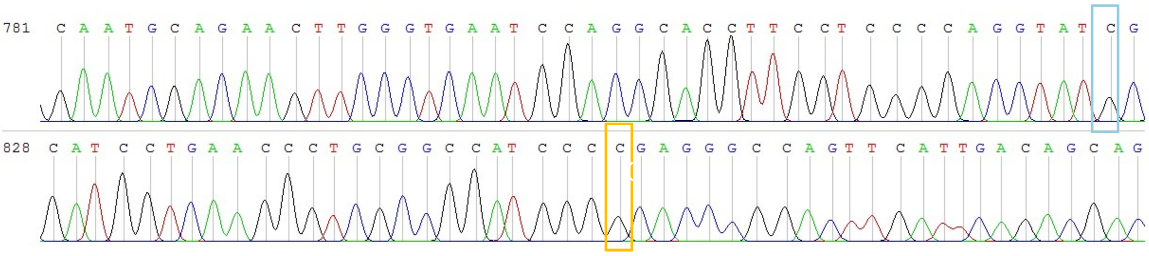

p1, both alles

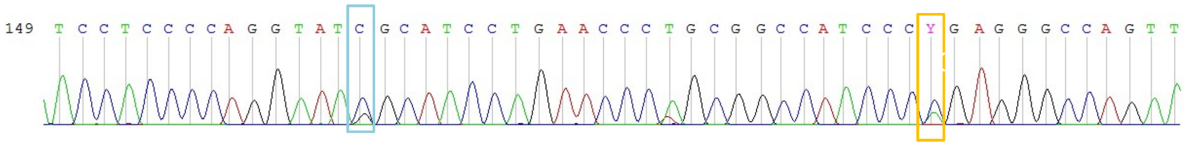

B

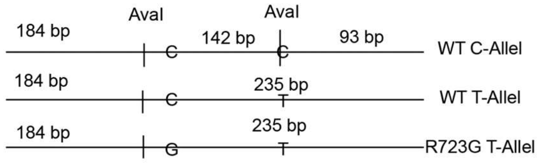

C

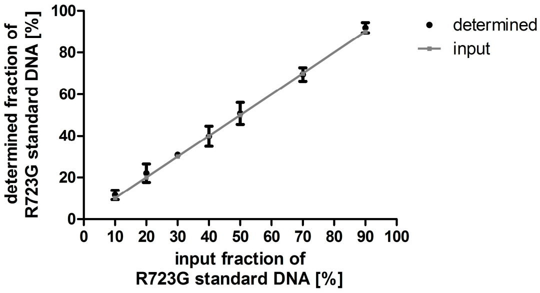

D

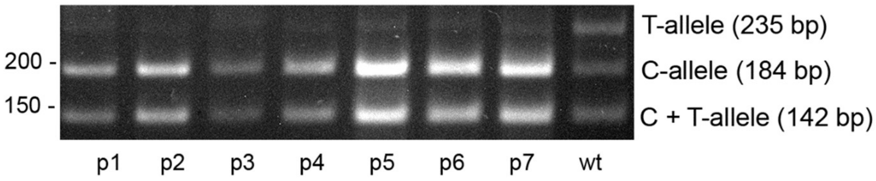

E

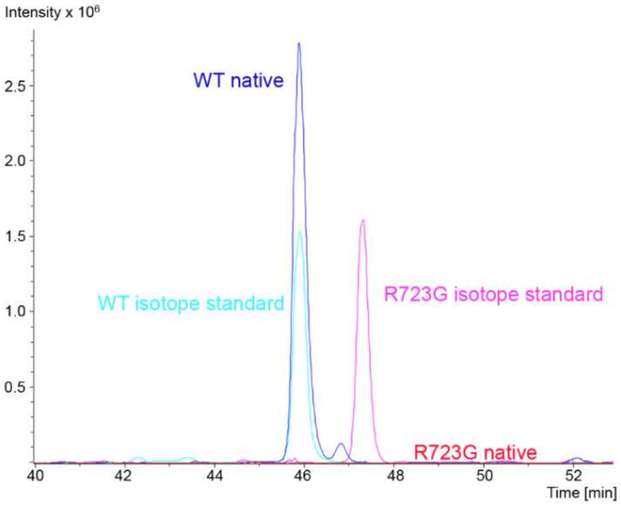

Figure S4

Figure S4: Relative quantification of *MYH7* alleles and ß-MyHC isoforms

(A) Using the primer pair Intron 18 for and Exon 20 rev (Table S1) the c.2193C-allele was exclusively amplified (compare: p1 (top panel) and p1 both alleles (lowest panel)). Thus this allele was PCR-amplified from genomic DNA of the seven genome edited piglets. PCR-products were subjected to Sanger sequencing. The nucleotide c.2193 is indicated by a yellow box, the nucleotide c.2167 (mutation site) by a blue box. **(B)** Schematic of restriction analysis for the relative quantification of the c.2193 C and T alleles. In the wildtype animals, both alleles encode for the C at position c.2167 (codon 723), however, upon *Ava*I restriction analysis both T- and C-allele can be discriminated. The genome edited animals encode for the wildype C at position c.2167 on the C-allele and for the mutated G at position c.2167 on the T-allele. *Ava*I restriction results in a common 184 bp fragment for both alleles. The c.2193 C-allele generates a 142 bp fragment and the c.2193 T-allele (R723G allele in the genome edited animals) generates a 235 bp fragment. **(C)** Defined ratios of standard plasmids for each allele were subjected to PCR and subsequent allele specific restriction analysis[7]. The restriction fragments were separated by agarose gel-electrophoresis and quantified densitometrically. The resulting integrated optical density (IOD) of each band was normalized to its length (bp). The respective fraction of each mixture was determined as the ratio of IOD/bp of each standard plasmid. **(D)** RT-PCR and subsequent AvaI-restriction was performed on RNA extracted from left ventricular/septum tissue of the seven genome edited animals and two natural breeding wildtype controls. A representative agarose gel analysis is shown. Note that even though the T-allele (R723G) specific band of 235 bp is substantially weaker in comparison to the C-allele in the genome edited animals, it is visible in each animal, indicating a biallelic expression of the *MYH7* gene. **(E)** Representative mass spectrometric quantification of WT and R723G ß-MyHC protein as described previously[8]. Stable-isotope labelled ß-MyHC peptides with mutation R723G and the corresponding wild-type peptide pair were spiked in equimolar ratios to myosin extracts from the genome edited animals and subjected to Lys-C treatment. NanoLC-ESI-MS separation was performed. Traces represent the ion signals of the 5-fold charged peptides. Isotope labelled synthetic mutant (magenta) and wild-type (turquois) peptides, and ion traces of native wild-type (blue) peptides were detected, whereas the signal for the native mutant peptide (red) was below the detection limit.

	MYH7	GAPDH
Slope	-3.333 ± 0.2026	-3.230 ± 0.2035
efficacy	1,00	1,04

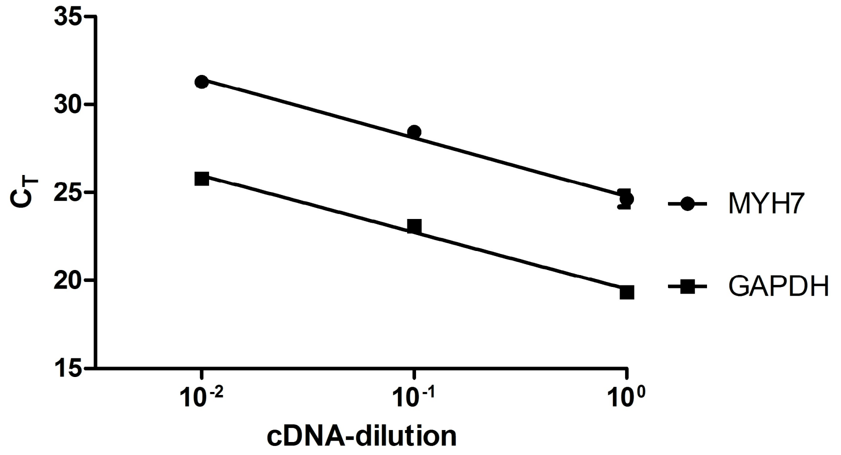

Figure S5. Relative quantification of *MYH7* and *GAPDH* mRNA. Total RNA was extracted from porcine cardiac tissue and mRNA was reverse transcribed using random hexamer primers. Dilutions of these cDNAs were subjected to real-time PCR analysis of *MYH7* and *GAPDH* mRNA expression. Efficacy of each primer set was calculated from the slope.

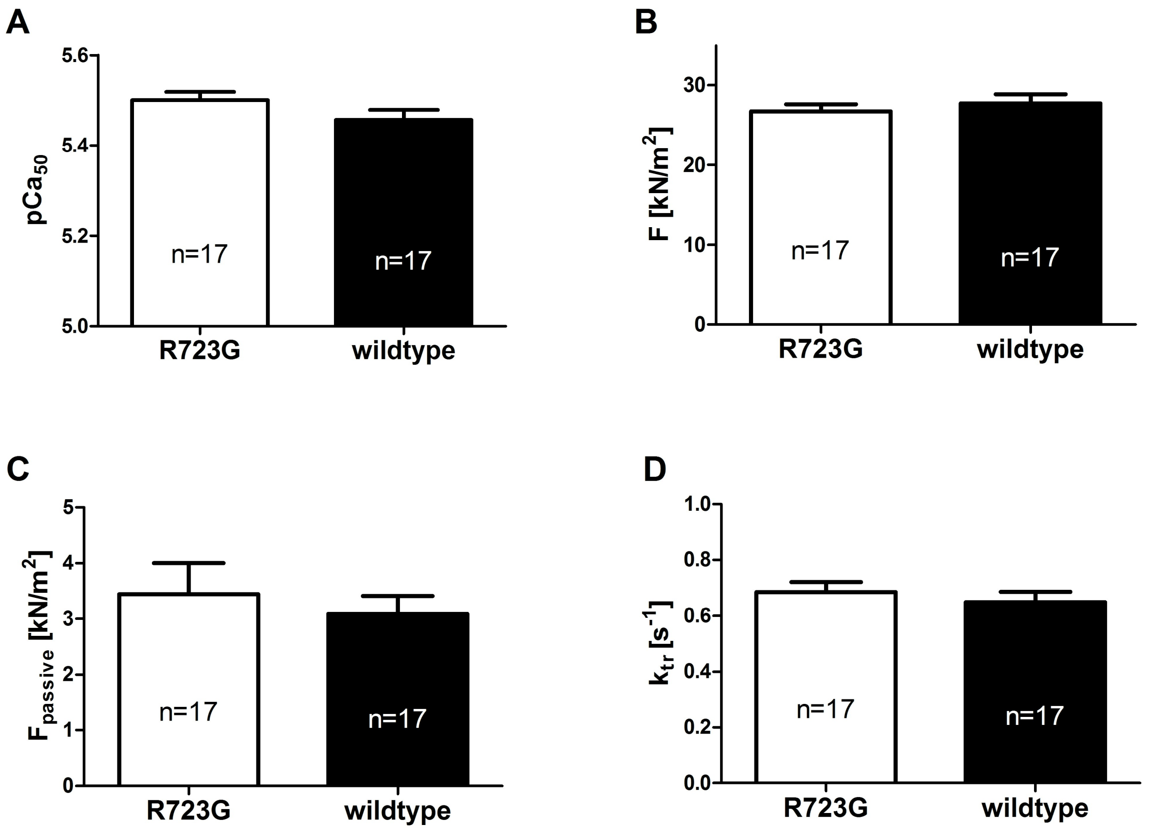

Figure S6: Functional analyses of single cardiomyocytes of the genome edited animals

Force measurements in cardiomyocytes Force measurements on single, mechanically isolated cardiomyocytes were performed as described previously[9]. In brief, single cardiomyocytes were isolated from left ventricular wall and septum of three genome edited animals (p5, p6, p7) and two age- and sex-matched controls (p8 and p9). Chemically permeabilized cardiomyocytes were attached to a motor and force transducer and incubated with protein kinase A (PKA) to adjust PKA-dependent phosphorylation levels. The cardiomyocytes were subjected to different calcium concentrations and force development under isometric conditions and the rate constant of force redevelopment after a quick release and restretch of the cardiomycoytes to isometric length (k_{tr}) were determined. The corresponding calcium concentration (given as pCa) at half maximum force (pCa_{50}) (A), maximum isometric force (B) and passive force per cross-sectional area (C), respectively, and the rate constant of force redevelopment (D) were determined for the genome edited and the wildtype controls. Mean and SEM of 17 single cardiomyocytes for each group are depicted.

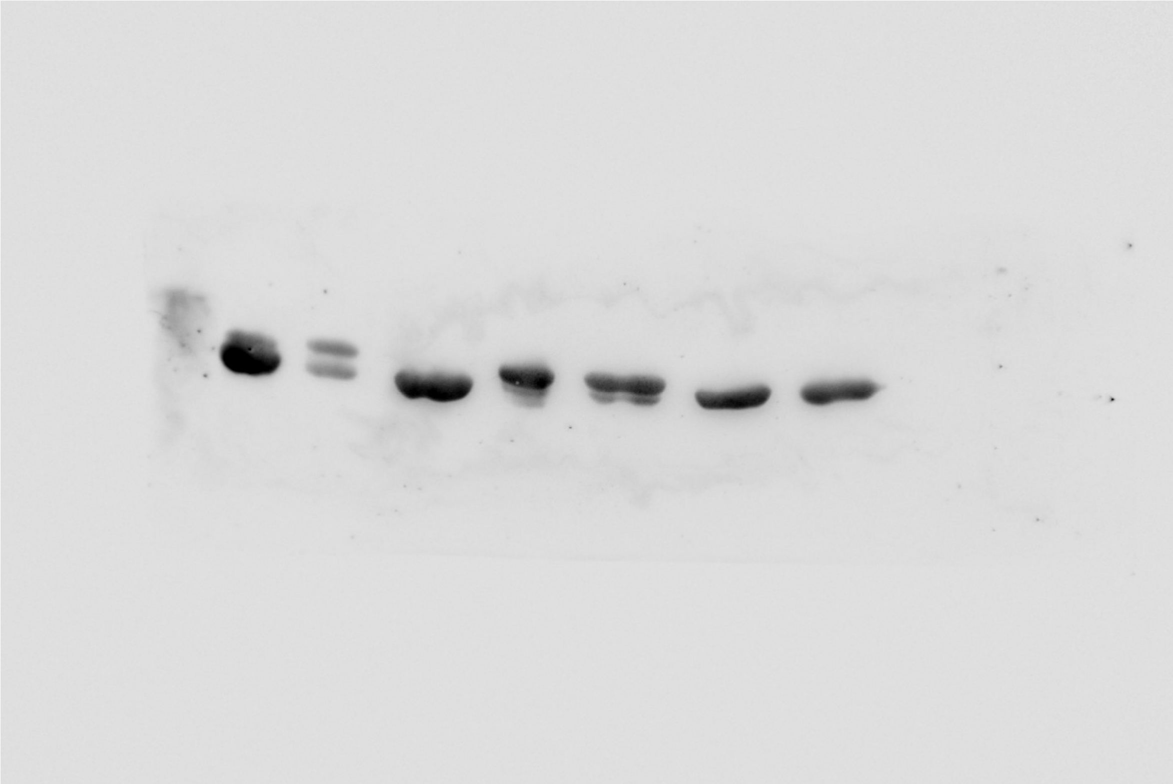

Figure S7: Full length blot of Figure 3D

References

1. Fananapazir, L., Dalakas, M. C., Cyran, F., Cohn, G. & Epstein, N. D. Missense mutations in the beta-myosin heavy-chain gene cause central core disease in hypertrophic cardiomyopathy. Proc Natl Acad Sci U S A. **90**, 3993-7 (1993).
2. Khosa, F. et al. Prevalence of non-cardiac pathology on clinical transthoracic echocardiography. J Am Soc Echocardiogr. **25**, 553-7 (2012).
3. Holub, A., Padalikova, D. & Filka, J. Glycogen in liver, heart and skeletal muscle of baby pigs in the early postnatal period. Veterinary Medicine. **6**, 201-208 (1961).
4. Lekanne Deprez, R. H. et al. Two cases of severe neonatal hypertrophic cardiomyopathy caused by compound heterozygous mutations in the MYBPC3 gene. J Med Genet. **43**, 829-32 (2006).
5. Weber, R. et al. Spectrum and outcome of primary cardiomyopathies diagnosed during fetal life. JACC Heart Fail. **2**, 403-11 (2014).
6. Nygard, A. B., Jorgensen, C. B., Cirera, S. & Fredholm, M. Selection of reference genes for gene expression studies in pig tissues using SYBR green qPCR. BMC Mol Biol. **8**, 67 (2007).
7. Tripathi, S. et al. Unequal allelic expression of wild-type and mutated beta-myosin in familial hypertrophic cardiomyopathy. Basic Res Cardiol. **106**, 1041-55 (2011).
8. Becker, E., Navarro-Lopez, F., Francino, A., Brenner, B. & Kraft, T. Quantification of mutant versus wild-type myosin in human muscle biopsies using nano-LC/ESI-MS. Anal Chem. **79**, 9531-8 (2007).
9. Kraft, T. et al. Familial hypertrophic cardiomyopathy: functional effects of myosin mutation R723G in cardiomyocytes. J Mol Cell Cardiol. **57**, 13-22 (2013).
